# Metformin‐Based Covalent Organic Frameworks With Excellent Biosafety and High Efficiency against Pathogenic Microorganisms

**DOI:** 10.1002/advs.202522437

**Published:** 2025-12-27

**Authors:** Jia‐Yi Liu, Hong Jiang, Lei Ma, Jin‐Yi Yu, Ming‐Yi Yang, Hao‐Ru Wang, Jun‐Yuang Tang, Shengfeng Huang, Wei Yi, Meng Lu, Ya‐Qian Lan, Xu‐Jia Hong

**Affiliations:** ^1^ Guangzhou Municipal and Guangdong Provincial Key Laboratory of Molecular Target & Clinical Pharmacology the NMPA and State Key Laboratory of Respiratory Disease School of Pharmaceutical Sciences Guangzhou Medical University Guangzhou P. R. China; ^2^ Guangdong Provincial Key Laboratory of Carbon Dioxide Resource Utilization School of Chemistry South China Normal University Guangzhou P. R. China; ^3^ The Affiliated Traditional Chinese Medicine Hospital Guangzhou Medical University Guangzhou P. R. China; ^4^ State Key Laboratory of Respiratory Disease National Clinical Research Center for Respiratory Disease Guangzhou Institute of Respiratory Health National Center for Respiratory Medicine The First Affiliated Hospital of Guangzhou Medical University Guangzhou P. R. China

**Keywords:** biosafety, covalent organic frameworks, metformin, photoactivity

## Abstract

The escalating public health security crisis caused by pathogenic microorganisms and the worsening antibiotic resistance urgently demand the development of antimicrobial materials with high efficiency and safety. Covalent organic frameworks (COFs) exhibit significant potential in photocatalytic antibacterial applications, but face challenges such as insufficient biosafety and difficulties in application translation. In this work, we first report two metformin‐based photoactive cationic MCOFs engineered for synergistic functionality and safety. They exhibit broad‐spectrum light absorption, efficient charge separation, and generate multiple ROS via Type I/II photodynamic, achieving >99 % inactivation of bacteria/viruses. Excellent biocompatibility with LD_50_ >5000 mg/kg is confirmed. Mechanistic studies based on molecular dynamics (MD) simulation and transcriptome results revealed a triple synergistic antibacterial pathway involving membrane targeting, ROS attack, and metabolic interference. Furthermore, we fabricated a COF/thermoplastic polyurethane (TPU) composite functional membrane, which exhibits >99 % antibacterial and antiviral efficiency, along with a notable 98.3 % inhibition rate against methicillin‐resistant Staphylococcus aureus (MRSA). This study not only provides a new design strategy for MCOFs with synergistically enhanced functionality and biosafety, but also offers a promising material platform for biomedical applications such as anti‐infection protection, wound healing, and the treatment of drug‐resistant bacterial infections.

## Graphical abstract

This work presents the first metformin‐based COFs featuring built‐in biosafety and a unique triple‐synergy mechanism. This scalable platform achieves broad‐spectrum eradication of bacteria and viruses, enabling reusable protective membranes with exceptional biocompatibility for advanced biomedical applications and personal protection.

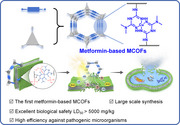

## 1 Introduction

Infectious diseases caused by pathogenic microorganisms such as bacteria and viruses continue to pose a persistent threat to global public health security [1, 2, 3]. The emergence of novel pathogens and the exacerbation of antibiotic resistance have made the development of highly efficient non‐pharmacological antimicrobial strategies that are less prone to inducing resistance particularly urgent [4, 5, 6, 7]. Photocatalytic technology, which utilizes light energy to generate reactive oxygen species (ROS) for microbial inactivation, has become a research hotspot due to its broad‐spectrum efficacy, high efficiency, environmental friendliness, and lower potential for inducing resistance [8, 9, 10]. However, traditional photocatalytic materials (e.g., inorganic semiconductor including TiO_2_, ZnO, CeO_2_, et al.) suffer from limitations such as low visible light utilization efficiency, insufficient ROS yield, and lack of biological targeting specificity, severely restricting their application in the biomedical field [11, 12, 13]. Therefore, it is essential to develop novel antibacterial and antiviral material systems to fulfill the above demands simultaneously. Additionally, precise structural design of the antimicrobial materials is also necessary to deeply elucidate the intrinsic action mechanism.

**SCHEME 1:**
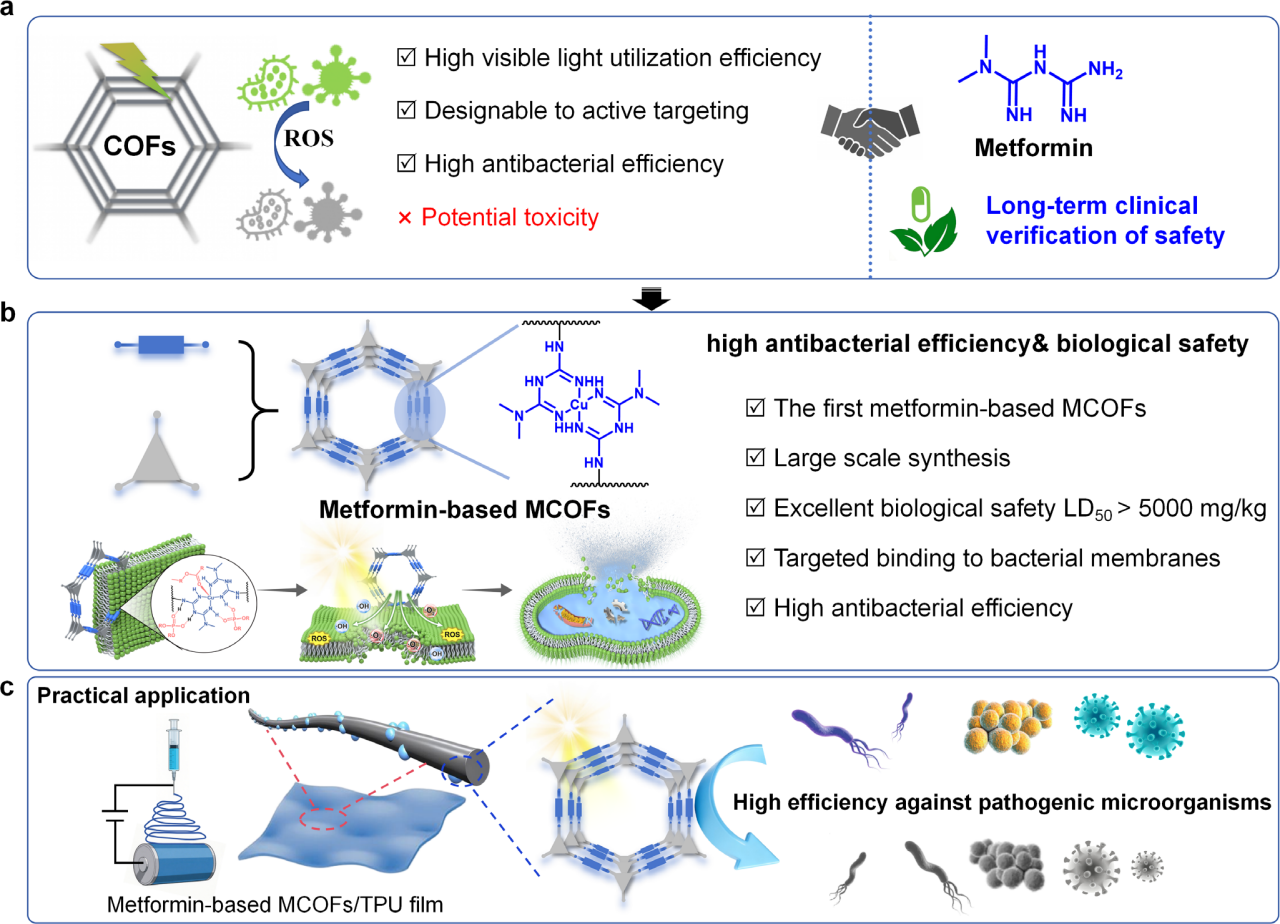
Schematic illustration of the design, synthesis, and application of metformin‐based MCOFs. (a) The current advantages and challenges of COFs in treating pathogenic microorganisms. (b) The design principle of metformin‐based COFs with high antibacterial efficiency& biological safety. (c) Metformin‐based MCOFs/TPU composite films for high efficiency against pathogenic microorganisms.

Covalent organic frameworks (COFs) showed remarkable potential in photocatalytic antibacterial and antiviral applications, owing to their excellent photophysical properties, precisely tunable pore structures, high specific surface area, and robust structural stability [14, 15, 16, 17]. In recent years, researchers have focused on designing COFs capable of efficiently generating reactive oxygen species (ROS) for pathogen inactivation [16, 18, 19, 20]. By leveraging the electronegative nature of microbial membranes, cationic COFs have been developed to enable targeted recognition and synergistic photocatalytic antibacterial, significantly enhancing antimicrobial efficiency [21, 22, 23, 24]. Moreover, the pre‐designable and functionally modifiable structures of COFs allow for the incorporation of biocompatible groups or drug molecules, offering substantial promise for biomedical applications [25, 26, 27]. However, current research on COFs remains predominantly focused on improving their photocatalytic antimicrobial performance, while systematic biosafety evaluation is often overlooked (Scheme 1a). This significantly limits the practical biological application of COFs. Thus, rational design COFs that are both highly antimicrobial and biosafe remain a crucial challenge.

The incorporation of clinically established drugs as building blocks for COFs represents a promising strategy to harmonize functionality and biosafety. Metformin, a widely used oral antidiabetic agent, is recognized for its favorable safety profile and biocompatibility (Scheme 1a) [28, 29, 30]. Recent evidence further indicates that metformin possesses intrinsic antibacterial activity and immunomodulatory functions, demonstrating inhibitory effects even against drug‐resistant strains [31, 32, 33]. However, as a small monomer molecule, the photoactivity of metformin relativity weak, thereby restricting its application in the field of photocatalytic antibacterial applications. If metformin is incorporated into the COFs backbone, three key advantages will be achieved simultaneous: (1) the COFs may inherit metformin's favorable pharmacological safety, which may provide an inherent biocompatibility foundation for the COF; (2) the photoactivity of the metformin‐based materials can be significantly enhanced; (3) metformin's cationic properties and potential membrane interaction mechanisms can endow the COF with the ability to actively target pathogenic microorganisms. Therefore, constructing COFs using metformin as a functional building block is expected to achieve synergistic enhancement of the material's biosafety and antibacterial function. It also provides a new material platform for COF applications in biomedical fields such as infection treatment, wound repair, and personal protection. However, it is still a great challenge to assemble COFs from metformin because the molecule itself does not contain multiple functional groups that enable further establish framework connection, which lead to the synthesis of metformin‐based COF structure has not been realized in this field so far. In this work, by coordinating metformin with copper ions to form a metal‐organic node (CuMet), we successfully designed a suitable building block with the symmetric amino groups for COF assembly via Schiff base reaction. Besides, the cationic CuMet showed significant contributions in the photoactivity, ROS generation, and antibacterial process.

Herein, for the first time, this study selected the clinically safe drug metformin as a functional building block to construct CuMet and then successfully assembled with aldehyde linkers under ambient temperature with ultrasonic assistance, efficiently synthesizing two novel metformin‐based MCOFs, CuMet‐TP COF and CuMet‐BTTH COF (Scheme 1b). On the one hand, inheriting the excellent biosafety of metformin, the metformin‐based MCOFs show negligible cytotoxicity (>75 % cell viability at 200 µg/mL) and high oral LD50 (>5000 mg/kg). On the other hand, molecular dynamics simulations and RNA sequencing reveal that the metformin‐based MCOFs can achieve membrane targeting and synergistic photocatalytic ROS generation via cationic CuMet units. They realized antibacterial activity through a triple synergistic pathway involving membrane targeting, photocatalysis, and metabolic interference. Under simulated sunlight irradiation, these MCOFs show extremely high inactivation efficiency (> 99 %) against both Gram‐positive and Gram‐negative bacteria. Their membrane materials can effectively inactivate broad‐spectrum pathogenic microorganisms such as *Escherichia coli* (*E. coli*), *Staphylococcus aureus* (*S. aureus*), methicillin‐resistant Staphylococcus aureus (MRSA), HCoV‐229E, and H9N2 with an activity of 99 %. The metformin‐based MCOFs developed in this work exhibit significant potential for various biomedical applications, due to their outstanding biosafety, highly efficient, and controllable antibacterial/antiviral properties.

## 2 Results

### 2.1 Synthesis and Characterization of Metformin‐Based MCOFs

**FIGURE 1:**
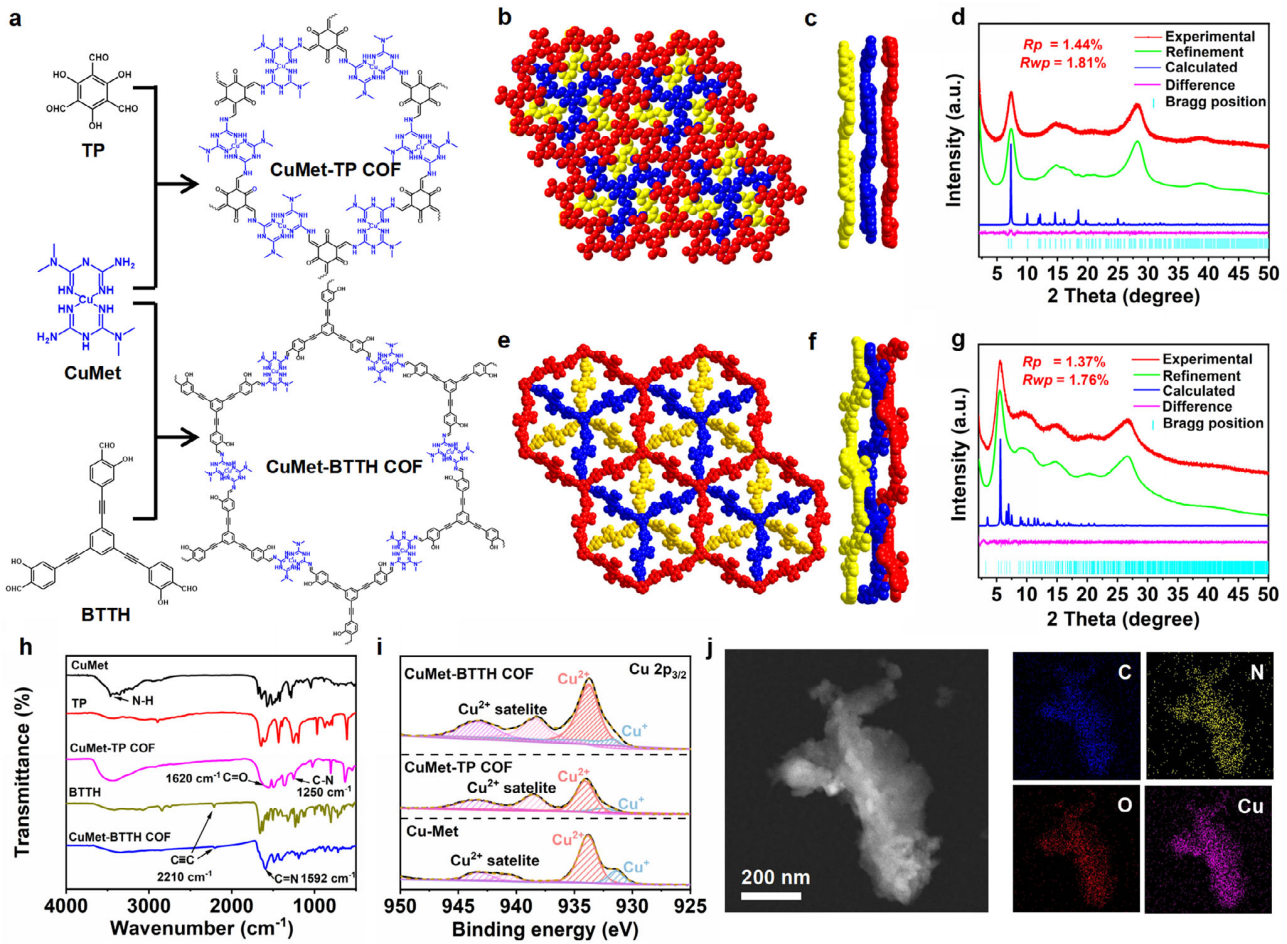
Synthesis and structural characterization of metformin‐based COFs. (a) Ultrasound‐assisted synthesis of CuMet‐TP COF and CuMet‐BTTH COF. (b,c) Simulated structure of CuMet‐TP COF (top and side views). (d) Pawley refinement of the PXRD patterns for CuMet‐TP COF. (e,f) Simulated structure of CuMet‐BTTH COF (top and side views). (g) Pawley refinement of the PXRD patterns for CuMet‐BTTH COF. (h) FT‐IR spectra of CuMet, TP, BTTH, CuMet‐TP COF, and CuMet‐BTTH COF. (i) High‐resolution Cu 2p3/2 XPS spectra of CuMet‐TP COF and CuMet‐BTTH COF. (j) Annular‐dark‐field EDX element mapping of CuMet‐TP COF.

As shown in Scheme 1b and Figure S1 for the crystal structure, metformin reacted with CuCl_2_ to form a planar complex CuMet. The amino groups retained at both ends of CuMet could further undergo condensation reactions with aldehyde monomers to assemble into metformin‐based COFs. Herein, for the first time, two novel metformin‐based MCOFs, CuMet‐TP COF and CuMet‐BTTH COF, were assembled through CuMet with TP and BTTH, respectively, under ambient temperature and ultrasonic assistance (Figure 1a). It's worth noting that this efficient synthesis method under ambient conditions with ultrasound assistance enabled rapid gram‐scale preparation of the metformin‐based MCOFs (Figure S2). The structure of CuMet‐TP COF was then simulated using the Materials Studio package. After structural optimization, the PXRD patterns were found to conform to the ABC stacking model (Figure 1b,c). The refinement results by using the Pawley method result in CuMet‐TP COF having unit cell parameters of a = b = 25.8615 Å and c = 14.4211 Å with Rwp = 1.81 %, Rp = 1.44 % (Figure 1d). The diffraction peaks at 7.31° and 14.62° were assigned to the (101) and (202) crystal planes, respectively. When the aldehyde monomer was extended to the larger BTTH, we further successfully formed CuMet‐BTTH COF, demonstrating the good suitability of CuMet as a linear connecting unit for MCOF construction. Simulation results showed that CuMet‐BTTH COF also crystallized in the ABC stacking mode (Figure 1e,f). Pawley refinement results (Figure 1g) revealed unit cell parameters of a = b = 50.5801 Å and c = 16.8219 Å (R*wp* = 1.76 %, R*p* = 1.37 %), with a diffraction peak at 5.52° corresponding to the (101) crystal plane.

Fourier transform infrared spectroscopy further confirmed the chemical structures of the two metformin‐based MCOFs (Figure 1h). For CuMet‐TP COF, a new characteristic peak appeared at 1250 cm^−1^, attributed to the C─N stretching vibration in the β‐ketoenamine structure, resulting from the typical enol‐keto tautomerism of the TP unit. For CuMet‐BTTH COF, the characteristic stretching vibration peak of the alkyne group remained at 2210 cm^−1^, while a peak for the Schiff base C═N stretching vibration appeared at 1592 cm^−1^, confirming its successful formation. N_2_ adsorption–desorption tests at 77 K showed low adsorption capacities in the micropore region for both MCOFs (Figure S3), consistent with pore constraints induced by their ABC stacking mode. X‐ray photoelectron spectroscopy analysis confirmed that the metformin‐based MCOFs are composed of C, N, O, and Cu elements (Figures S4 and S5). High‐resolution Cu 2p_3/2_ spectra revealed that Cu in both CuMet‐TP COF and CuMet‐BTTH COF exists in a mixed +1 and +2 valence state (Figure 1i). As shown in Figures S6–S9, scanning electron microscopy (SEM) and transmission electron microscopy (TEM) images displayed a flake‐like nanomaterial morphology for the metformin‐based COFs. Elemental mapping images (Figures 1j; S10) further confirmed the uniform distribution of C, N, O, and Cu elements within the materials. Additionally, Zeta potential measurements indicated that the synthesized CuMet‐TP COF and CuMet‐BTTH COF maintained the positive charge characteristic of CuMet (Figure S11), confirming them as cationic framework materials. These multiple characterization results consistently demonstrate the successful construction of metformin‐based MCOFs with well‐defined crystal structures and chemical compositions.

### 2.2 Photocatalytic Mechanism and Antimicrobial Activity of Metformin‐Based MCOFs

**FIGURE 2:**
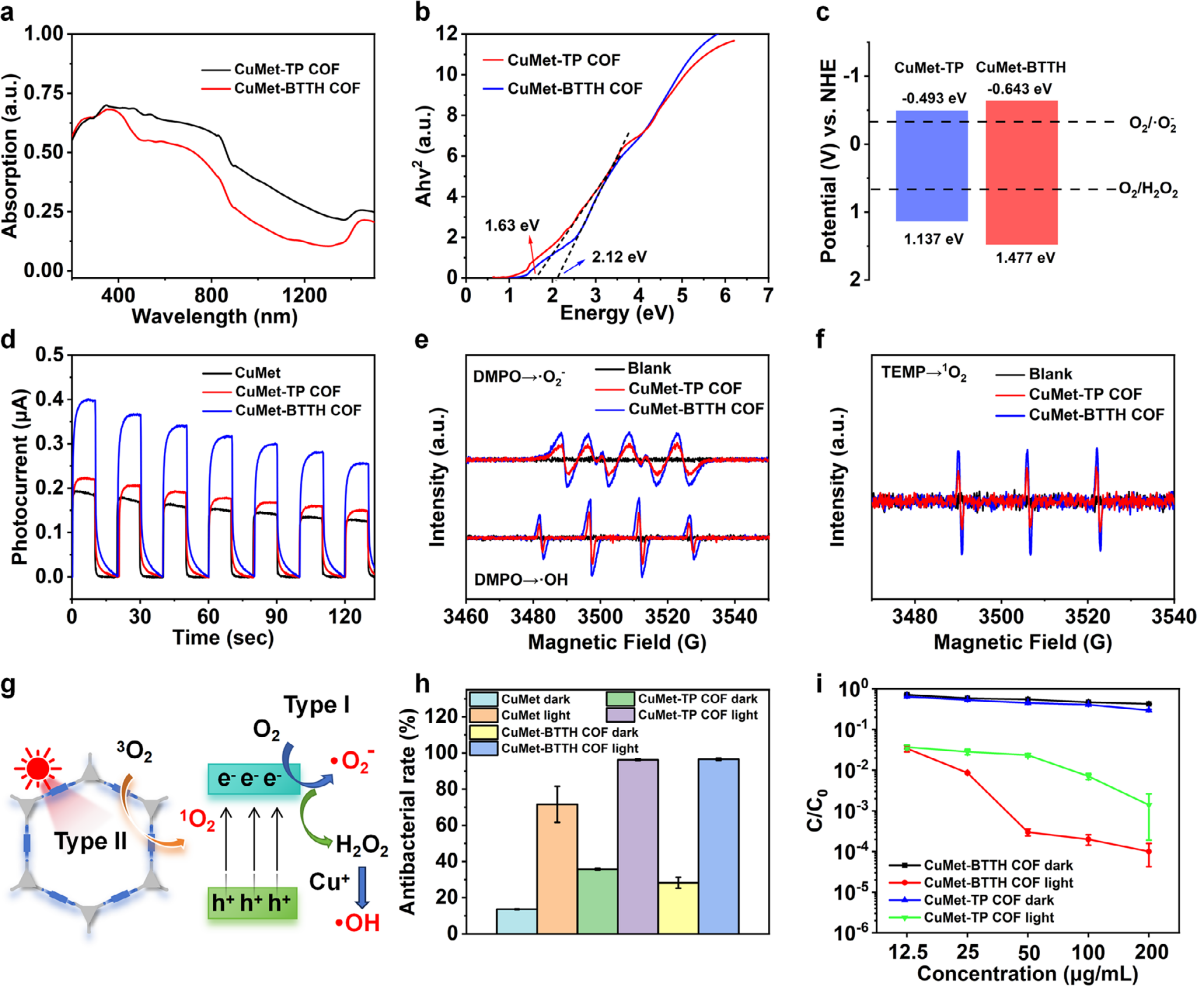
Photocatalytic performance and antibacterial activity of metformin‐based COFs. (a) UV–vis–NIR absorption spectra and (b) Tauc plots for band gap estimation results of CuMet‐TP COF and CuMet‐BTTH COF. (c) Schematic of band structure and ROS generation potentials. (d) Transient photocurrent response curves of CuMet, CuMet‐TP COF, and CuMet‐BTTH COF. (e,f) EPR spectra under light illumination: (e) DMPO‐•O_2_^−^, and DMPO‐•OH, (f) TEMP‐^1^O_2_. (g) The photocatalytic ROS generation mechanism of reactive oxygen species by metformin‐based COFs. (h) Antibacterial rates against *E. coli* for different materials at 12.5 µg/mL. (i) Antibacterial performance of CuMet‐TP COF and CuMet‐BTTH COF against *E. coli* under light and dark conditions at various concentrations.

UV–vis absorption spectroscopy analysis revealed that CuMet‐TP COF and CuMet‐BTTH COF exhibit broad absorption characteristics across the 200–1500 nm range (Figure 2a), indicating excellent light‐harvesting ability and potential for solar‐driven photocatalysis. To evaluate their photodynamic therapy (PDT) performance, the optical band gaps (Eg) were calculated from Tauc plots, yielding values of 1.63 and 2.12 eV for CuMet‐TP COF and CuMet‐BTTH COF, respectively (Figure 2b). Mott–Schottky measurements performed at frequencies between 3000 and 5000 Hz showed positive slopes for both materials within the tested potential range (Figures S12 and S13), characteristic of n‐type semiconductors. Based on band position calculations, the conduction band (CB) positions of CuMet‐TP COF and CuMet‐BTTH COF were −0.493 and −0.643 eV (vs. NHE), respectively, while their valence band (VB) positions were 1.137 and 1.477 eV (vs. NHE), respectively. The band structure diagram (Figure 2c) indicates that both materials possess the ability to photocatalytically reduce O_2_ to •O_2_^−^ and H_2_O_2_. To further investigate photogenerated carrier behavior, photoluminescence spectra were measured (Figure S14). Under 350 nm excitation, CuMet, CuMet‐TP COF, and CuMet‐BTTH COF all exhibited an emission peak at 437 nm, with fluorescence intensity following the order: CuMet > CuMet‐BTTH COF > CuMet‐TP COF. The lower fluorescence intensity suggests suppressed recombination of photogenerated electron–hole pairs in the COF structures, attributable to their highly ordered conjugated skeletons promoting charge separation and migration. This result is consistent with transient photocurrent response tests (Figure 2d), where both CuMet‐TP COF and CuMet‐BTTH COF exhibited stronger photocurrent responses than CuMet, confirming the beneficial role of the COF structure in separating and transporting photogenerated charge carriers.

To clarify the generation of ROS in the photocatalytic reaction, 3,3',5,5'‐tetramethylbenzidine (TMB) oxidation experiments were conducted. As shown in Figures S15 and S16, under light irradiation, the TMB solution exhibited a significant color reaction with increasing COF concentration, while no change was observed in the dark, preliminarily proving photocatalytic ROS generation. Electron paramagnetic resonance (EPR) technology was further used to identify ROS species. 5,5‐dimethyl‐1‐pyrroline N‐oxide (DMPO) was used to trap •O_2_^−^ and •OH, and 2,2,6,6‐tetramethylpiperidine (TEMP) was used to trap ^1^O_2_. As shown in Figure 2e–g, characteristic signals for DMPO‐•O_2_^−^, DMPO‐•OH, and TEMP‐^1^O_2_ were clearly detected after illumination, indicating the effective production of all three ROS types. From the band structure perspective (Figure 2c), the conduction band potentials of the COFs are sufficient to reduce O_2_ to •O_2_^−^ (‐0.33 V vs. NHE). Although the valence band potentials are insufficient to directly oxidize H_2_O to •OH (+2.68 V vs. NHE), H_2_O_2_ can be generated via stepwise O_2_ reduction (−0.67 V vs. NHE), which can then be converted to •OH through a Fenton‐like reaction catalyzed by Cu⁺ active sites. Simultaneously, the detected ^1^O_2_ indicates that the materials can also undergo a Type II photodynamic reaction via an energy transfer mechanism. The above results demonstrate that metformin‐based COFs possess a synergistic Type I/II photodynamic mechanism, enabling the simultaneous generation of •O_2_^−^, •OH, and ^1^O_2_ under light irradiation. Among them, CuMet‐BTTH COF exhibits stronger ROS generation capacity due to its superior charge separation efficiency (Figure 2e–g). In addition, XRD tests indicated that the two metformin‐based COFs exhibited excellent light stability and pH stability, laying a solid foundation for their subsequent applications (Figure S17).

Based on the efficient ROS generation ability of metformin‐based MCOFs, we further evaluated their antibacterial performance against *E. coli* and *S. aureus*. Under simulated sunlight irradiation (110 mW/cm^2^), the metformin‐based COFs exhibited significantly better antibacterial activity than the CuMet monomer (Figures 2h; S18). At a concentration of 12.5 µg/mL, both CuMet‐TP COF and CuMet‐BTTH COF achieved over 96 % inactivation of *E. coli*, whereas CuMet only reached 71.61 %. At 50 µg/mL, the inactivation rates for S. aureus reached 98.4 % and 99.56 % for the two COFs, significantly higher than the 81.59 % achieved by CuMet. Dose‐effect and light comparison experiments further showed (Figures 2i; S19–S21) that antibacterial activity increased limitedly with concentration in the dark, while light irradiation significantly enhanced the antibacterial effect, with CuMet‐BTTH COF exhibiting the best performance. At a concentration of 200 µg/mL under 30 min of light irradiation, the inactivation rates of CuMet‐TP COF and CuMet‐BTTH COF against both bacteria reached over 99 %, demonstrating excellent broad‐spectrum photocatalytic antibacterial performance.

### 2.3 Investigation of the Antimicrobial Mechanism of Metformin‐Based MCOFs

**FIGURE 3:**
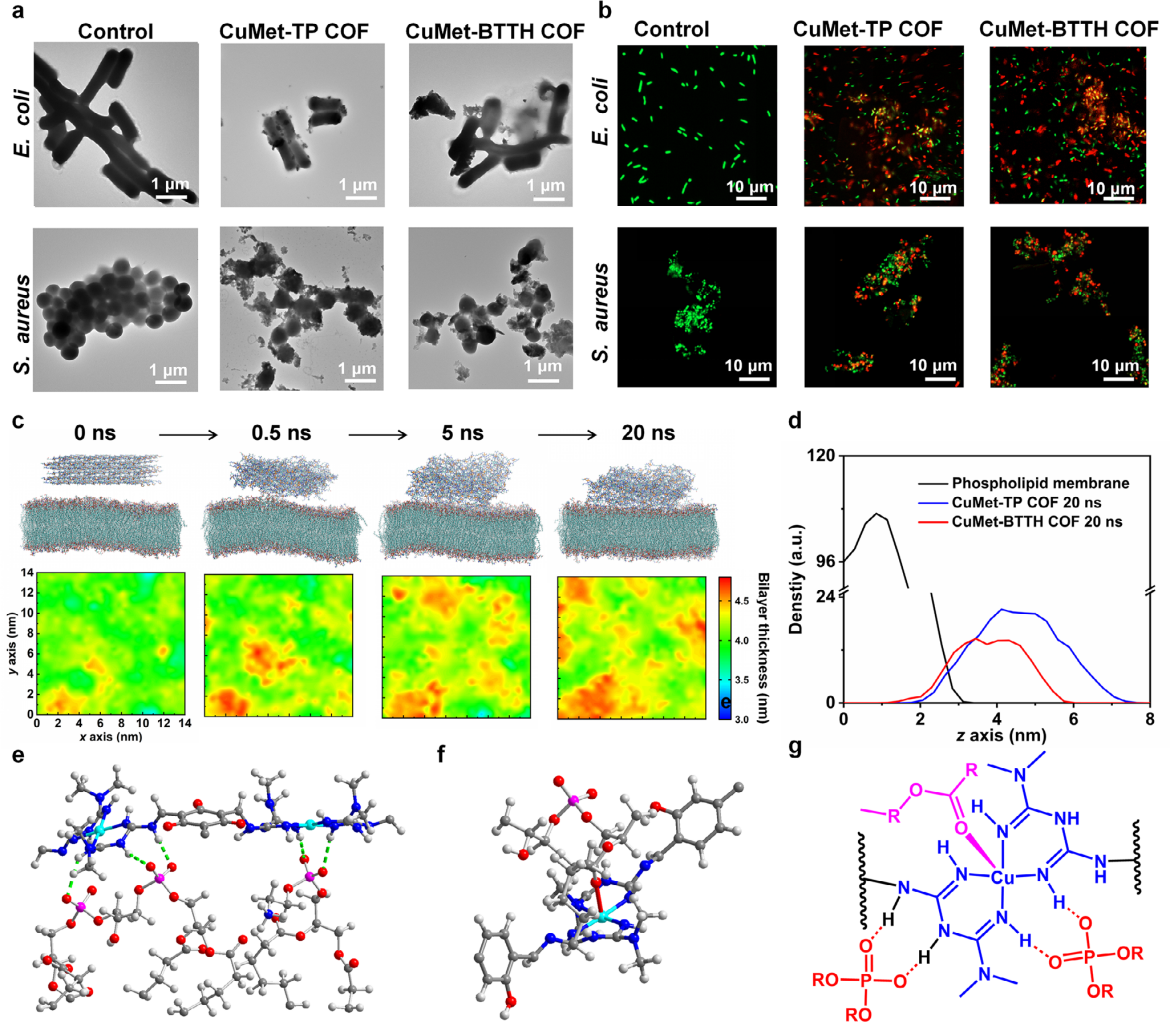
Antibacterial mechanism of metformin‐based COFs (cellular and molecular characterization). (a) TEM images of bacteria after interaction with COFs, showing membrane rupture and material adhesion. (b) Fluorescence images of *E.coli* and *S.aureus* stained with AO (green, live bacteria) and PI (red, dead bacteria) after various treatments. (c) Molecular dynamics simulation process of interaction between CuMet‐TP COF and *E.coli* bacterial phospholipid membrane within 20 ns. (d) Longitudinal distribution density curves of CuMet‐TP COF and CuMet‐BTTH COF on bacterial phospholipid membrane at 20 ns. (e) The intermolecular hydrogen bond interaction between CuMet‐TP COF and the phospholipid membrane at 20 ns. (f) The coordination interaction between CuMet‐BTTH COF and phospholipid membranes at 20 ns. (g) The interaction mode between the CuMet unit in metformuan‐based COFs and the phospholipid groups in the phospholipid membrane.

To explore the antimicrobial mechanism of the metformin‐based MCOFs, the morphological changes of bacteria after interaction with CuMet‐TP COF and CuMet‐BTTH COF were observed. The SEM and TEM images show that both *E. coli* and *S. aureus* treated with the two MCOFs exhibited significant morphological distortions, including cell twisting, shrinkage, and local membrane folding, rupture, or even collapse (Figures 3a; S22). Clear adhesion of MCOF materials to the bacterial surface was observed, indicating interactions between the MCOFs and bacterial membranes. To further evaluate bacterial viability, live/dead staining experiments were performed using DMAO/PI fluorescent dyes. Confocal laser scanning microscopy (CLSM) images showed (Figure 3b) that bacteria in the control group only exhibited green fluorescence, indicating intact membranes and unaffected activity. In the experimental group treated with MCOFs material and light, large areas of red fluorescence were observed, indicating that the bacterial membrane was damaged, allowing PI to enter the cells to label nucleic acids. This result reconfirms the excellent photocatalytic antibacterial performance of metformin‐based MCOFs from the perspective of cell viability. Additionally, bacterial regrowth experiments showed (Figures S23 and S24) that the OD_600_ value of the control group increased significantly from 0.1 to over 0.8 after 2 days of culture, indicating normal proliferation. In contrast, the OD_600_ values of the experimental groups treated with metformin‐based MCOFs showed almost no change, indicating inhibited bacterial proliferation and further confirming the effective bactericidal ability of these materials.

Combining the results of morphological observation, live/dead staining, and regrowth experiments, it is clear that CuMet‐TP COF and CuMet‐BTTH COF exhibit significant antibacterial effects under light irradiation. We propose that their antibacterial mechanism mainly stems from two aspects. First, the cationic metformin‐based MCOFs can target and bind to the negatively charged bacterial membrane through electrostatic interactions, causing initial membrane damage. Second, under light irradiation, the MCOFs efficiently generate ROS, which further exacerbate membrane lipid peroxidation, protein denaturation, and DNA damage, ultimately leading to cell death. This synergistic mechanism of active targeting, binding, and photocatalytic ROS killing provides a crucial basis for their high‐efficiency antibacterial performance.

To delve deeper into this mechanism at the molecular level, we employed molecular dynamics (MD) simulations and transcriptome sequencing (RNA‐seq) analysis to study the interaction between COFs and bacterial membranes and their regulatory effects on bacterial gene expression. The complete MD simulation process of the complex system of metformin‐based COFs and bacterial membranes is shown in Figures 3c and S25. CuMet‐TP COF and CuMet‐BTTH COF rapidly interacted with the phospholipid membrane surface within 0.5 ns and stably associated, gradually embedding deeper into the membrane over time. The 2D plots of average membrane thickness at different time points (Figures 3c; S25) showed increasing variation in thickness distribution over time. After 20 ns, the disturbance of the phospholipid membrane by the MCOFs reached its maximum, indicating that both metformin‐based COFs could rapidly adsorb and stably bind to the phospholipid membrane surface. The longitudinal density distribution curves of the COFs on the membrane at 20 ns also showed that both were completely embedded within the membrane (Figure 3d). To further elucidate the interactions between the MCOFs and the phospholipid membrane, we performed quantitative analysis of the binding energy based on the MD simulations. Both CuMet‐TP COF and CuMet‐BTTH COF exhibited substantial Coulombic and van der Waals interactions with the membrane, confirming a spontaneous binding process (Figures S26, S27). The binding affinity increased progressively over the simulation time. At 0.5 ns, the calculated binding energies were −162.34 kJ/mol for CuMet‐TP COF and −314.46 kJ/mol for CuMet‐BTTH COF. These values increased to −1962.4 kJ/mol and −2336.89 kJ/mol at 5 ns, respectively, and reached their maxima at 20 ns, at −6553.8 kJ/mol and −5367.1 kJ/mol, respectively. The above results all indicate strong and primarily spontaneous interactions between metformin‐based MCOFs and phospholipid membranes.

Notably, analysis of binding conformations revealed that the CuMet units in metformin‐based COFs are the main sites for binding to the phospholipid membrane. As shown in Figures S28 and S29, at the initial 0.5 ns, obvious hydrogen bonding interactions occurred between the ─NH groups from the CuMet units of the MCOFs and the phospholipid headgroups. It is worth noting that the Cu atoms from the CuMet units of CuMet‐BTTH COF also formed coordination interactions with ester oxygen atoms. Figures 3e,f, and S30 further illustrate the binding modes of metformin‐based MCOFs after full contact with the phospholipid membrane at 20 ns. Abundant hydrogen bonding interactions were observed between the ─NH groups from the CuMet units of the MCOFs and the oxygen atoms from the phosphate groups in the phospholipid membrane. The coordination between Cu and ester oxygen persisted in CuMet‐BTTH COF. In summary, as depicted in Figure 3g, metformin‐based MCOFs can utilize their CuMet units to bind to the phospholipid headgroups of the membrane through hydrogen bonding and coordination bonding. Simultaneously, the cationic regions provided by the metformin ligands significantly enhance electrostatic attraction with the negatively charged bacterial membrane, thereby disrupting membrane stability.

**FIGURE 4:**
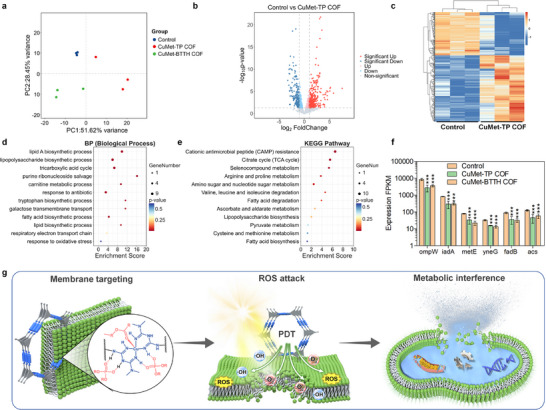
Transcriptome analysis of antibacterial mechanism and summary of synergistic mechanism for metformin‐based COFs. (a) PCA of gene expression profiles from *E. coli* treated with CuMet‐TP COF, CuMet‐BTTH COF, and a control group. (b) Volcano plot showing differentially expressed genes in *E. coli* after CuMet‐TP COF treatment. (c) Hierarchical clustering heatmap of 390 differentially expressed genes between the CuMet‐TP COF treatment group and control. (d) GO enrichment analysis of biological processes significantly affected by CuMet‐TP COF treatment. (e) KEGG pathway enrichment analysis. (f) Expression levels of key genes. Mean ± SD (*n* = 3).^***^ *p*< 0.001. (g) Schematic illustration of the proposed triple synergistic antibacterial mechanism of membrane targeting, ROS attack, and metabolic interference.

The membrane binding mechanism revealed at the molecular level provides a critical physicochemical context for interpreting RNA‐seq data. To systematically analyze the antibacterial molecular mechanism of CuMet‐TP COF and CuMet‐BTTH COF, RNA‐seq technology was used to profile the transcriptome expression of *E. coli* treated with the two materials (Figure 4). Principal component analysis (PCA) showed clear separation between the control group and the two treatment groups (Figure 4a), indicating that the metformin‐based MCOFs significantly reshaped the bacterial gene expression patterns. Volcano plot analysis further identified numerous differentially expressed genes (DEGs). Compared with the control group, the CuMet‐TP COF group had 154 genes significantly downregulated and 236 upregulated (Figure 4b), while the CuMet‐BTTH COF group had 85 downregulated and 197 upregulated (Figure S31a). Cluster heatmaps clearly displayed the expression trends of DEGs between groups, with 390 DEGs between CuMet‐TP COF and control (Figure 4c), and 282 between CuMet‐BTTH COF and control (Figure S31b).

Gene Ontology (GO) and Kyoto Encyclopedia of Genes and Genomes (KEGG) enrichment analyses (Figures 4d,e; S31c,d) showed that the DEGs were significantly enriched in several key functional pathways, primarily falling into three categories: (1) Membrane biosynthesis‐related processes (e.g., lipid and lipopolysaccharide biosynthesis, galactose metabolism, fatty acid degradation); (2) Oxidative stress response and detoxification pathways (e.g., selenium compound metabolism, cysteine and methionine metabolism); (3) Energy metabolism processes (e.g., TCA cycle, branched‐chain amino acid degradation). These pathways were overall significantly inhibited. Gene expression analysis (Figures S32 and S33; Figure 4f) showed that both materials caused the downregulation of several core genes, including membrane structure‐related genes (e.g., ompW) and key energy metabolism genes (e.g., fadB and acs). These results indicate that the metformin‐based COFs simultaneously disrupt outer membrane integrity and impede energy generation, leading to physical barrier collapse and energy depletion.

Combining MD simulation and transcriptome results, we propose that metformin‐based COFs achieve high antibacterial efficiency through a synergistic amplification cycle composed of membrane targeting, ROS attack, and metabolic interference (Figure 4g). This cycle is initiated by the electrostatic targeting and binding of the cationic metformin‐copper unit to the bacterial membrane, providing a platform for subsequent close‐range assault. Under light irradiation, the in situ‐generated ROS directly induce membrane lipid peroxidation, exacerbating membrane damage and increasing permeability. The compromised membrane, in turn, facilitates the enhanced penetration of ROS and possibly material components into the cell, leading to more effective inhibition of key metabolic pathways and resulting in oxidative stress and biosynthetic disruption. The collapse of metabolic function further cripples the cellular repair machinery, rendering the bacterium more vulnerable to the initial membrane damage and ongoing ROS attack. Thus, membrane damage, ROS attack, and metabolic interference are not isolated events but form a self‐reinforcing vicious cycle. Each process exacerbates the next and is, in turn, amplified by the subsequent effects, ultimately driving irreversible bacterial death. This multi‐target synergistic mechanism effectively circumvents the resistance bottlenecks associated with conventional antimicrobials and offers novel insights for designing next‐generation antibacterial materials.

### 2.4 Biosafety of Metformin‐Based COFs and Protective Performance of Membrane Materials

**FIGURE 5:**
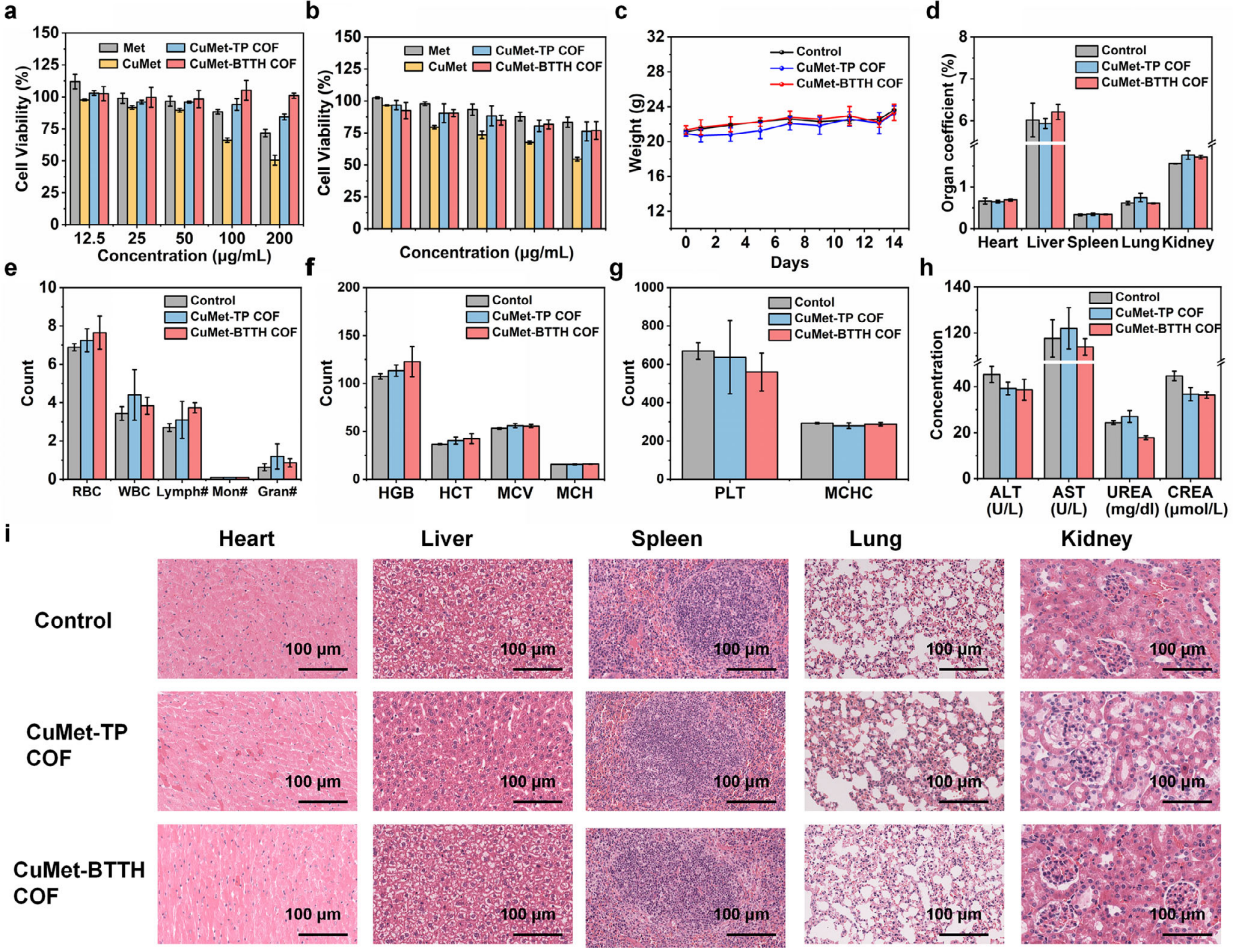
In vitro and in vivo biosafety evaluation of CuMet‐TP COF and CuMet‐BTTH COF. (a) Viability of L929 cells after treatment with different concentrations of CuMet‐TP COF and CuMet‐BTTH COF. (b) Viability of BEAS‐2B cells after treatment with different concentrations of the COFs. (c) Body weight changes of mice in the control group and the COFs‐treated groups within 14 days. (d) Organ/body weight ratios of heart, liver, spleen, lung, and kidney. (e, f, g) Complete blood count analysis: red blood cell count (RBC), white blood cell count (WBC), lymphocyte count (Lymph#), monocyte count(Mon#), granulocyte count (Gran#), hemoglobin (HGB), hematocrit (HCT), mean corpuscular volume (MCV), mean corpuscular hemoglobin (MCH), platelet count(PLT), and mean corpuscular hemoglobin concentration(MCHC). (h) Serum biochemical parameters: liver function markers‐alanine aminotransferase (ALT) and aspartate aminotransferase (AST); renal function markers‐urea nitrogen (UREA) and creatinine (CREA). (i) H&E‐stained sections of major organs from mice after different treatments.

Given the excellent antimicrobial performance of CuMet‐TP COF and CuMet‐BTTH COF, we further systematically evaluated their biosafety to assess potential risks in practical applications. Cytotoxicity assays (Figure 5a,b) showed that at 200 µg/mL, the relative survival rates of L929 and BEAS‐2B cells treated with both MCOFs were above 75 %, indicating negligible cytotoxicity.

To further evaluate their safety in vivo, acute toxicity tests were conducted. After oral gavage administration at 5000 mg/kg, no mortality or behavioral abnormalities were observed in mice treated with either CuMet‐TP COF or CuMet‐BTTH COF over a 14‐day observation period. Body weight changes showed no significant difference compared to the control group (Figure 5c). Notably, key hematological and serum biochemical parameters, such as liver function markers (ALT, AST) and renal function markers (UREA, CREA), in both the CuMet‐TP COF and CuMet‐BTTH COF groups fell within normal ranges and showed no statistically significant differences compared to the control group (Figure 5d–h; Table S4). Furthermore, H&E staining of vital organs (heart, liver, spleen, lung, kidney) revealed no obvious histological lesions (Figure 5i). These in vitro and in vivo safety assessments consistently demonstrate the good biosafety of the metformin‐based MCOF materials. This not only confirms the advantage of using clinically safe drugs as structural units for designing and tuning MCOF biocompatibility but also provides a crucial safety basis for their practical application in biomedical fields such as antibacterial dressings and protective coatings.

**FIGURE 6:**
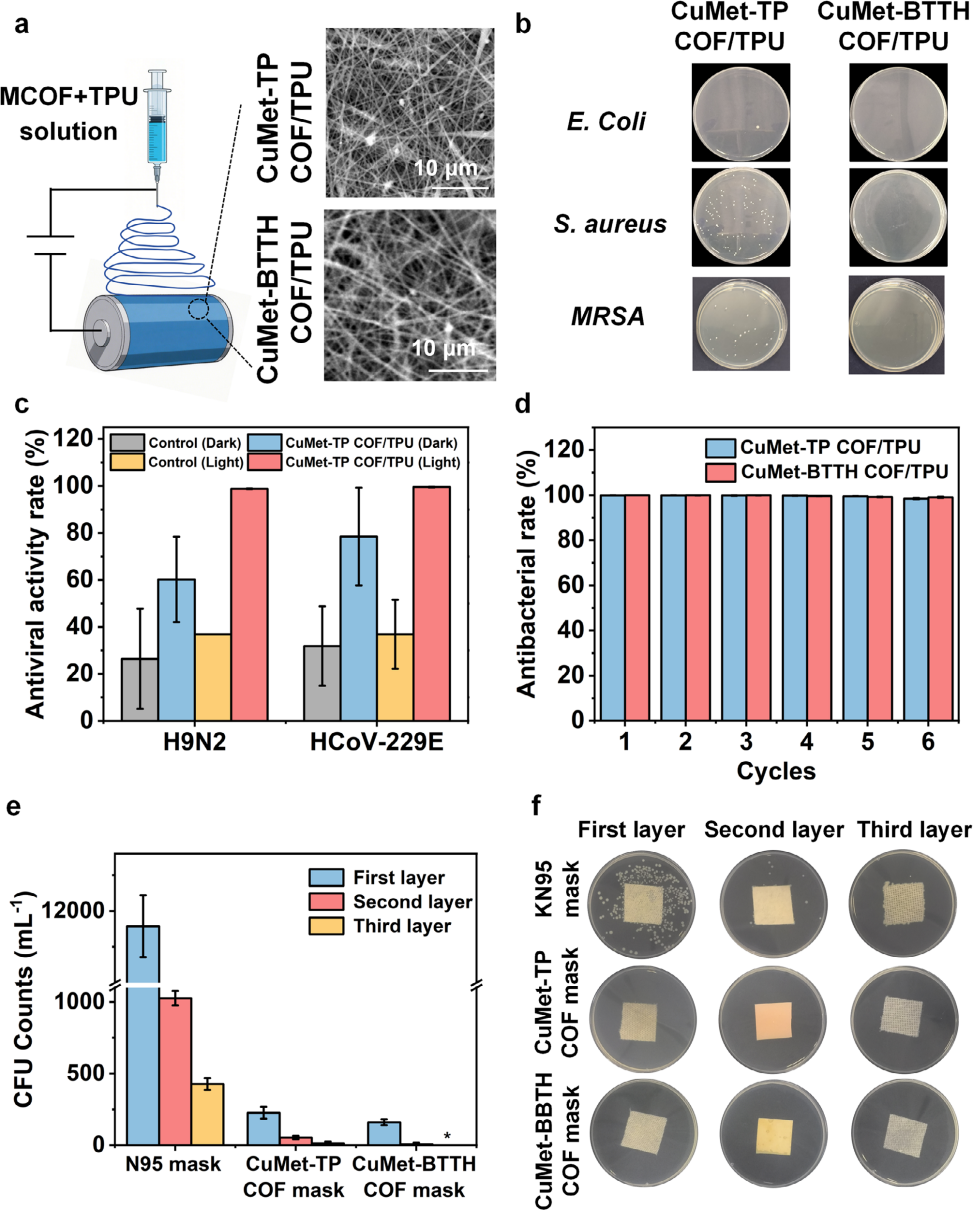
Application of metformin‐based COFs against pathogenic microorganisms and personal protective equipment. (a) Schematic diagram of preparing metformin‐based COFs/TPU composite films by electrospinning (inset: SEM images of CuMet‐TP COF/TPU and CuMet‐BTTH COF/TPU composite films). (b) Photographs of *E.coli*, *S. aureus*, and *MRSA* colonies formed on LB agar plates after photocatalytic antibacterial treatment of different COF/TPU composite membrane materials. (c) Photocatalytic antiviral activity rate of CuMet‐TP COF/TPU composite film. (d) *E. coli* inactivation rates of CuMet‐TP COF/TPU and CuMet‐BTTH COF/TPU electrospun membranes after 6 cycles of reuse. (e,f) The residual levels of *E.coli* on the first, second, and third layers of the commercial N95 mask, CuMet‐TP COF/TPU mask, and CuMet‐BTTH COF/TPU mask (* in (d) denotes the undetectable level of CFU mL^−1^ counts).

Based on their excellent antimicrobial activity and biosafety, we further demonstrated the potential biomedical applications potential of metformin‐based MCOFs. Using electrospinning technology, CuMet‐TP COF and CuMet‐BTTH COF were compounded with TPU to successfully prepare functional fibrous membranes with uniformly loaded MCOFs (Figure 6a). SEM images showed good dispersion of the COFs within the fibers. Then, we systematically evaluated the performance of the COF/TPU composite membranes against various pathogenic microorganisms. As shown in Figure 6b, under simulated sunlight, the COF/TPU composite membranes exhibited significant antibacterial effects against both Gram‐negative bacteria (*E. coli*) and Gram‐positive bacteria (*S. aureus*). Specifically, the CuMet‐TP COF/TPU membrane showed a high antibacterial rate of >99 % against *E. coli*, while the CuMet‐BTTH COF/TPU membrane also achieved >99 % inhibition against both *E. coli* and *S. aureus*. Notably, the membranes demonstrated a remarkable inhibition rate of 98.3 % against *MRSA*, indicating their potential value in addressing the crisis of antibiotic resistance. Notably, the material exhibited exceptional reusability, maintaining an antibacterial rate above 99 % after six consecutive cycles (Figures 6d; S34). Furthermore, using CuMet‐TP COF/TPU as a representative, we evaluated its antiviral capability. It is shown that the material achieved inactivation rates of 98.83 % and 99.57 % against H9N2 and HCoV‐229E, respectively (Figure 6c), indicating broad‐spectrum anti‐pathogen capability. These results suggest its strong potential for protecting against various aerosol‐transmitted pathogens.

Inspired by these results, we successfully integrated the COF/TPU composite membrane as a middle filtration layer into masks to construct protective masks with photocatalytic self‐cleaning functionality (Figure S35). For comparison, a commercial N95 mask, which relies solely on physical filtration without any intrinsic antimicrobial activity, was used as the control. After exposure to an aerosol environment containing *E. coli* and 30 min of light irradiation, nearly no live bacteria were detected in the second and third layers of the COF‐based mask, whereas a substantial number of bacteria remained on the corresponding layers of the commercial N95 mask (Figures 6e,f; S36). The results indicate that the metformin‐based COF fibrous membrane can significantly enhance the active protective performance of masks, superior to traditional masks relying solely on physical filtration. In addition, to better simulate realistic application environments, we evaluated the antibacterial performance of masks assembled with CuMet‐TP COF/TPU and CuMet‐BTTH COF/TPU composite membranes under both low and high humidity conditions. As demonstrated in Figure S37, the masks based on these composite membranes maintained highly efficient protective performance even under varying humidity levels. These results collectively provide strong support for the practical application potential of our materials in diverse and changing real‐world conditions.

While the results highlight the potential of our metformin‐based MCOFs for applications such as protective masks, future efforts to further introduce stimuli‐responsive functionalities into this material platform could significantly broaden its utility, particularly in more complex biomedical scenarios. For instance, enabling the material to respond to a broader range of physiological stimuli in addition to light would allow for spatiotemporally controlled, on‐demand antibacterial activity, akin to advanced smart systems reported recently [34, 35, 36, 37, 38]. Such an evolution would address potential limitations in deep‐tissue or low‐light environments while building upon the excellent biosafety and photocatalytic foundation established here. This direction promises to advance our material toward next‐generation, adaptive, and intelligent antibacterial applications.

## 3 Conclusion

In summary, this work has successfully realized the pioneering design and construction of metformin‐based COFs by employing a clinically safe drug as the functional building block. This strategy effectively reconciles the often‐conflicting demands of high antimicrobial potency and excellent biosafety in photocatalytic materials. The synthesized CuMet‐TP COF and CuMet‐BTTH COF inherit the outstanding biocompatibility of metformin, as unambiguously demonstrated by negligible cytotoxicity and a high acute oral LD_50_ exceeding 5000 mg/kg. Meanwhile, they also achieve broad‐spectrum pathogen inactivation efficiency exceeding 99 % against both *E. coli* and *S. aureus* under simulated sunlight, through a triple synergistic mechanism of membrane targeting, ROS attack, and metabolic interference. Beyond powder materials, we demonstrated significant practical potential by fabricating COF/TPU composite membranes via electrospinning. These membranes retained the superior biosafety and broad‐spectrum against pathogenic microorganisms of the COFs, exhibiting remarkable performance against drug‐resistant bacteria like MRSA (98.3 % inhibition) and maintaining high efficiency over multiple reuse cycles. The successful integration of these membranes into protective masks, providing enhanced, active defense against bioaerosols, highlights a promising translational pathway. This research thus delivers a versatile and biocompatible material platform, offering a novel design paradigm for developing advanced antimicrobial materials. It addresses the critical challenge of biosafety in nano‐antibacterial agents and opens up new avenues for applications in personal protective equipment, wound management, and the fight against drug‐resistant infections.

## 4 Experimental Section

### 4.1 Synthesis of CuMet‐TP COF and CuMet‐BTTH COF

For CuMet‐TP COF, CuMet (235.9 mg,0.6 mmol) and sodium p‐toluenesulfonate (116.5 mg, 0.6 mmol) were dissolved in deionized water (20 mL) to form solution A. 2,4,6‐triformylphloroglucinol (TP) (84 mg, 0.4 mmol) was dissolved in chloroform (30 mL) as solution B. After 10 min of ultrasonic pretreatment, solutions A and B were combined and subjected to ultrasonic homogenization for 1 h. The resulting products were collected and washed twice each with deionized water and ethanol. For CuMet‐BTTH COF, CuMet (117.9 mg,0.3 mmol) and sodium p‐toluenesulfonate (116.5 mg, 0.6 mmol) were dissolved in deionized water (20 mL) to form solution A. 4,4′,4″‐(benzene‐1,3,5‐triyltris(ethyne‐2,1‐diyl))tris(2‐hydroxybenzaldehyde) (BTTH) (102 mg, 0.2 mmol) was dissolved in chloroform (30 mL) as solution B. After 10 min of ultrasonic pretreatment, solutions A and B were combined and subjected to ultrasonic homogenization for 1 h. The resulting products were collected and washed twice each with deionized water and ethanol.

### 4.2 In Vivo Acute Oral Toxicity and Biosafety Evaluation of Metformin‐Based MCOFs

The acute oral toxicity of metformin‐based MCOFs was tested according to GB 15670‐1995. Female and male specific pathogen‐free (SPF) BALB/c mice (8–10 weeks old) were purchased from Zhuhai Bestest Biotechnology Co., Ltd. (license No. SYXK‐2023‐0186). All experimental protocols were approved by the Animal Ethics Committee of Guangzhou Yongnuo Medical Laboratory (IACUC Approval No. IACUC‐AEWC‐F241226022). After 1 week of adaptive feeding, mice were randomly divided by gender and body weight into 3 groups (n = 10 per group): control, CuMet‐TP COF, and CuMet‐BTTH COF. Following a 12 h fast (with water withheld), mice in the treatment groups received a single intragastric administration of the respective COFs at 5000 mg/kg (20 mL/kg), while the control group received an equal volume of normal saline. General conditions, including mental status, activity, food intake, and mortality, were monitored for 14 days, with body weight recorded every two days. At the endpoint, blood samples were collected for hematological and biochemical analyses. Major organs (heart, liver, spleen, lung, and kidney) were excised, weighed for organ coefficient calculation, and subjected to gross observation and histopathological examination using H&E staining.

### 4.3 Statistical Analysis

All quantitative data are presented as mean ± standard deviation (SD). Unless otherwise specified, the sample size for each experiment was n = 3. Potential outliers were evaluated for all datasets, but none were excluded. Statistical analyses were performed using GraphPad Prism software (GraphPad Software, San Diego, CA, USA). Significant differences between groups were assessed using Student's *t*‐test (for two‐group comparisons) or one‐way ANOVA (for multi‐group comparisons). All tests were two‐sided. The significance levels were defined as ^*^ *p*< 0.05, ^**^ *p*< 0.01, ^***^ *p*< 0.005, and ^****^ *p*< 0.001.

## Author Contributions

X.‐J.H., Y.‐Q.L., J.‐Y.L., and M.L. conceived the idea. J.‐Y.L., H.J., and L.M. designed the experiments, collected and analyzed the data. J.‐Y.Y., M.Y., H.‐R.W., J.‐Y.T., S.H., and W.Y. assisted with the experiments and characterizations. X.‐J.H. and J.‐Y.L. wrote the manuscript. All authors have approved the final version of the manuscript.

## Conflicts of Interest

The authors declare no conflict of interest.

## Supporting information




**Supporting File**: advs73557‐sup‐0001‐SuppMat.pdf.

## Data Availability

The data that support the findings of this study are available from the corresponding author upon reasonable request.
