# Supplementary material for: Metformin‐Based Covalent Organic Frameworks With Excellent Biosafety and High Efficiency against Pathogenic Microorganisms
Source: Adv Sci (Weinh). 2025 Dec 27;13(14):e22437. doi: 10.1002/advs.202522437 (PMC12970249; doi:10.1002/advs.202522437)
Supplement: Supplementary file 1 — Supporting File: advs73557‐sup‐0001‐SuppMat.pdf. [file ADVS-13-e22437-s001.pdf]

Supplemental information

Metformin-Based Covalent Organic Frameworks with Excellent Biosafety and High Efficiency Against Pathogenic Microorganisms

Jia-Yi Liu,[1,‡] Hong Jiang,[1,‡] Lei Ma,[1,‡] Jin-Yi Yu,[1] Ming-Yi Yang,[2] Hao-Ru Wang,[4] Jun-Yuang Tang,[1] Shengfeng Huang,[1] Wei Yi,[1] Meng Lu,[2,] Ya-Qian Lan,[2,]* and Xu-Jia Hong[1,3,]**

[1]Guangzhou Municipal and Guangdong Provincial Key Laboratory of Molecular Target & Clinical Pharmacology, the NMPA and State Key Laboratory of Respiratory Disease, School of Pharmaceutical Sciences, Guangzhou Medical University, Guangzhou 511436, China. E-mail: hongxujia@gzhmu.edu.cn (X.-J. H.)

[2] Guangdong Provincial Key Laboratory of Carbon Dioxide Resource Utilization, School of Chemistry, South China Normal University, Guangzhou, 510006, P. R. China. E-mail: yqlan@m.scnu.edu.cn (Y.-Q. L.), menglu@m.scnu.edu.cn (M. L.)

[3]The Affiliated Traditional Chinese Medicine Hospital, Guangzhou Medical University, Guangzhou 511436, China.

[4]State Key Laboratory of Respiratory Disease, National Clinical Research Center for Respiratory Disease, Guangzhou Institute of Respiratory Health, The First Affiliated Hospital of Guangzhou Medical University, National Center for Respiratory Medicine, Guangzhou 510120, P. R. China.

[‡]Jia-Yi Liu, Hong Jiang and Lei Ma contributed equally to this work.

S1. Material and Characterization methods

Materials

All solvents and reagents obtained from commercial sources were used without further purification. Copper (II) chloride dihydrate ($CuCl_2·2H_2O$) was purchased from Bide Pharmatech Co., Ltd (China). Metformin hydrochloride ($C_4H_{11}N_5·HCl$) was purchased from Shanghai Acmec Biochemical Technology Co., Ltd (China). Sodium p-toluenesulfonate (TsNa) was purchased from Shanghai Adamas Reagent Co., Ltd (China). Sodium hydroxide (NaOH), sodium sulfate (Na_2SO_4), reduced glutathione (GSH), tetramethylbenzidine (TMB) and hydrogen peroxide (H_2O_2, 30%) were purchased from Shanghai Aladdin Bio-Chem Technology Co., Ltd (China). 2,4,6-Triformylphloroglucinol (TP), 4,4',4''-(Benzene-1,3,5-triyltris(ethyne-2,1-diyl)) tris (2-hydroxybenzaldehyde) (BTTH) were purchased from Shanghai Tensus Biotech Co., Ltd (China). Cell Counting Kit-8 (CCK-8) and LIVE/DEAD Bacterial Viability Kit with DMAO and PI were purchased from Beyotime Biotechnology Co., Ltd (China). Luria-Bertani (LB) broth was purchased from Biosharp, a brand of Beijing Labagic Technology Co., Ltd (China). LB agar (No. 028330) was purchased from Guangdong Huankai Microbial Sci.&Tech. Co., Ltd (China). PBS, Dulbecco's modified Eagle's medium (DMEM), trypsin-EDTA (0.25%), fetal bovine serum (FBS), and antibiotics (penicillin-streptomycin, PS) were purchased from Gibco Life Technologies. Nafion was purchased from DuPont de Nemours, Inc (America). Ketjenblack was purchased from Guangdong Canrd New Energy Technology Co., Ltd. 5,5-Dimethyl-1-pyrroline N-oxide (DMPO) and 2,2,6,6-Tetramethyl-4-piperidone hydrochloride (TEMP) were purchased from Dojindo Laboratories (Japan). RNA-prep pure Cell/Bacteria Kit (DP430) and TIAN Seq-rRNA Depletion Kit were purchased from TIANGEN BIOTECH (BEIJING) CO., LTD (China). VAHTS Universal V6 RNA-seq Library Prep was purchased from Vazyme Biotech Co., Ltd (China). Dichloromethane (DCM) and ethanol were purchased from Guangzhou Bebetter Medicine Technology Co., Ltd (China).

Characterization

The powder X-ray diffraction (PXRD) patterns were recorded on a Bruker D8 Advance with Cu Kα radiation (λ = 1.5418 Å) at 40 kV, 40 mA. Diffraction intensity data for 2θ from 2° to 50° were collected at the scanning speed of 2 deg/min with a 2θ step increment of 0.01°. The single crystal diffraction (XRD) test was performed using the Bruker D8 Venture X-ray single crystal diffractometer (Mo/Cu target and liquid metal Ga target). The nanomaterials were characterized by field emission scanning electron microscope (Thermo Apreo 2C, equipped with an energy-dispersive spectrometer Oxford Ultim Max65) and field emission transmission electron microscope (Talos-F200s). The surface chemical properties of the samples were determined by X-ray photoelectron spectroscopy (XPS, Thermo Nexsa). The nitrogen adsorption-desorption isotherms were determined by BET specific surface area and pore size analyzer (Micromeritics ASAP 2460). Fourier transform infrared

spectroscopy (FT-IR) was acquired using a Thermo Fisher-Nicolet iS20 Fourier transform infrared spectrometer. The zeta potential of the sample was measured by Zetasizer Nano ZS90. The UV-visible absorption spectrum of the liquid was obtained by Shimadzu UV-vis spectrophotometer (model UV 3600I plus), while the solid UV-visible absorption spectrum was obtained by UV-Visible near-infrared spectrophotometer (model PerkinElmer Lambda1050+). The detection wavelength range was 200-1500 nm, and the bandgap was explored. The photoluminescence (PL) spectra were obtained using an Edinburgh Instruments FLS 1000 transient steady-state spectrometer with a detection wavelength range of 250-800 nm.

Synthesis and Single-Crystal Preparation of CuMet

An aqueous solution of $CuCl_2{\cdot}2H_2O$ (4.7 mmol, 800 mg) in 2 mL deionized water was slowly added to a vigorously stirred aqueous solution of metformin hydrochloride (4.0 mmol, 663 mg) and NaOH (4.8 mmol, 192 mg) in 10 mL deionized water. After stirring for 10 min, the resulting precipitate was collected by centrifugation and subjected to vacuum freeze-drying to obtain CuMet. For single-crystal preparation, a portion of the as-synthesized CuMet powder (20 mg) was dissolved in 4 mL of methanol. Purple rectangular crystals suitable for X-ray diffraction were obtained after slow evaporation of the solvent at room temperature over two days (CCDC No. 2487526).

S2. Photocatalysis and Antimicrobial Microorganism Experiments

Electrochemical characterization

Electrochemical characterization tests were performed in a standard three-electrode system of the CHI660E electrochemical workstation. In the experiment, platinum was used as the counter electrode, Ag/AgCl was used as the reference electrode, and the electrolyte was 0.1 M sodium sulfate solution. Among them, the Mott-Schottky test experiment is tested by the Impedance-Potential (IMPE) method. The transient photocurrent response test under periodic visible light on/off irradiation conditions was tested by the Amperometric i-t Curve method, and the test samples were tested in different states, that is, in simulated sunlight and darkness, the state switching time was 10 s.

Photocatalytic oxidation of TMB experiment

The 2 mmol/L TMB solution was prepared, and then different concentrations of CuMet-TP COF material and CuMet-BTTH COF material (0-200 μg/mL) were prepared with normal saline, and 200 μM TMB solution was added. After Shaking well, it was irradiated under simulated sunlight (110 mW/cm^2) for 30 min. After the reaction, it was centrifuged and detected by ultraviolet-visible spectrophotometer at 350-800 nm (with the characteristic peak at 650 nm). At the same time, the same operation was performed with only 200 μg/mL material under dark conditions as the control group.

Reactive oxygen species detection

The types of reactive oxygen species were detected by a Bruker EMXplus paramagnetic resonance spectrometer (EPR). Hydroxyl radicals and superoxide radicals were captured by 5,5-dimethyl-1-pyrroline N-oxide (DMPO), and singlet oxygen was captured by 2,2,6,6-tetramethyl-4-piperidone hydrochloride (TEMP).

Evaluation of Antibacterial Activity of COFs

Escherichia coli (ATCC 25922, *E. coli*) and *Staphylococcus aureus* (ATCC 25923, *S. aureus*) were obtained from Microbiologics (USA). Bacteria were grown on LB agar medium containing 10 g/L tryptone, 5 g/L NaCl, 1.0 g/L glucose, 5 g/L yeast extract, 15.0 g/L agar (pH 7.0 ± 0.2) at 37 ° C. *E.coli* and *S.aureus* were cultured in LB agar at 37 °C for 18-24 h, respectively. A single colony was resuspended in sterile saline 0.9% (w/v), and the optical density was adjusted to an OD_{600} of 0.10 ± 0.01 for *E.coli* or 0.08 ± 0.01 for *S.aureus*, corresponding to approximately 10^7 CFU/mL after gradient dilution. For antibacterial test, CuMet-TP COF or CuMet-BTTH COF (12.5-200 μg) was added to 1 mL of bacterial solution (10^7 CFU/mL) and irradiated under simulated sunlight (300 W xenon lamp with AM1.5 filter, 110 mW/cm^2) for 30

min. The bacterial concentration was then determined by plate counting after incubation at 37 °C for 18 hours. The antibacterial rate was calculated based on colony-forming unit (CFU) using Equation (1):

$$\text{Antibacterial rate}=\frac{C_0\text{-}C}{C_0}\times 100\% \tag{1}$$

Among them, C represents the CFU value of the experimental group after treatment of the bacterial solution, and C_0 represents the CFU value of the untreated control group.

In the absence of light or materials, the control experiment was performed according to the same steps. All data represent mean ± standard error from three independent measurements.

SEM characterization and TEM characterization for bacterial samples

The bacterial suspension treated with 200 μg/mL CuMet-TP COF or CuMet-BTTH COF under 30-minute illumination was washed with sterile saline and centrifuged at 8000 rpm for 5 minutes. The bacterial cells were fixed with 2.5% glutaraldehyde overnight, then centrifuged again at 8000 rpm for 10 minutes, washed, and subjected to gradient dehydration using ethanol series(20%, 40%, 60%, 80%, and 100%, 10 minutes each). A saline-treated group without COFs served as the control and was processed identically. Finally, the samples were sputter-coated with gold and imaged by SEM and TEM.

Bacterial dead/live viability assays

Bacterial suspension treated with 200 μg/mL of either CuMet-TP COF or CuMet-BTTH COF under 30-minute illumination were washed with sterile saline and centrifuged at 8000 rpm for 5 minutes. The bacterial cells were stained using a live/dead bacterial viability kit (DMAO/PI) according to the manufacturer's instructions. DMAO stains viable bacteria with intact membranes green, whereas propidium iodide (PI) enters bacteria with compromised membranes, staining dead cells red. A control group was prepared by replacing the COF solution with normal saline, followed by the same processing steps. After staining, the samples were imaged under a laser scanning confocal microscope using a 100× oil immersion objective.

RNA sequencing and analysis of bacterial samples

E. coli suspension was irradiated with 200 μg/mL CuMet-TP COF or CuMet-BTTH COF for 30 min, and then 1 mL of the liquid was transferred to 100 mL of LB broth and incubated in a shaker (220 rpm, 37 °C) for 5 h. Bacterial cells were harvested by centrifugation (3000 rpm, 4 °C, 5 min), washed with sterile water, and total RNA was extracted using an RNAprep Pure Cell/Bacteria Kit following the manufacturer's

instructions. RNA purity and concentration were measured on a NanoDrop 2000 spectrophotometer, and integrity was assessed using an Agilent 2100 Bioanalyzer. Ribosomal RNA was removed with the TIANSeq rRNA Depletion Kit. Sequencing libraries were prepared with the VAHTS Universal V6 RNA-seq Library Prep Kit. Raw FASTQ reads were quality-filtered and adapter-trimmed using Trimmomatic. High-quality reads were aligned to the reference genome with Rockhopper2, and gene expression levels were quantified in FPKM. Differential expression analysis was performed using DESeq2, with significantly differentially expressed transcripts defined as those exhibiting $|\log_2(\text{fold change})| > 1$ and an adjusted p-value < 0.05. Functional enrichment analysis of GO and KEGG terms was conducted via a hypergeometric test.

Cell Culture and Cytotoxicity Assessment of materials

BEAS-2B (human bronchial epithelial cells) and L929 (mouse fibroepithelial cells) were obtained from the Cell Bank of the Chinese Academy of Sciences (Shanghai, China). BEAS-2B cells were cultured in DMEM supplemented with 10% fetal bovine serum (FBS) and 1% penicillin-streptomycin. L929 cells were maintained in MEM containing 10% FBS and 1% penicillin-streptomycin. Both cell were incubated at 37 °C in a 5% CO_2 atmosphere. For cytotoxicity evaluation, L929 and BEAS-2B cells were treated with metformin, CuMet, CuMet-TP COF and CuMet-BTTH COF, respectively. Cells were seeded in 96-well plates at a density of 1×10^4 cells per well and cultured for 24 h to allow attachment. Subsequently, the medium was replaced with 200 μL of fresh medium containing nanomaterials at concentrations ranging from 0 to 200 μg/mL, followed by incubation at 37 °C and 5% CO_2 for 24 h. After treatment, the cells were washed twice with PBS and then incubated with 200 μL of serum-free medium containing 10% CCK-8 reagent at 37 °C for 2 h. The absorbance at 450 nm was measured using a microplate reader, and the cell viability was calculated by Equation (2):

$$\text{Cell Viability (\%)} = \frac{A_s - A_{b2}}{A_c - A_{b1}} \times 100\% \qquad (2)$$

Among them, A_s is the absorbance of the sample well (cells + medium + CCK-8 + drug), A_c is the absorbance of the control well (cells + medium + CCK-8), A_{b1} is the absorbance of the background well (medium + CCK-8), and A_{b2} is the absorbance of the material background well(medium + CCK-8 + drug).

Preparation of CuMet-TP COF/TPU and CuMet-BTTH COF/TPU Composite Membranes via Electrospinning

CuMet-TP COF or CuMet-BTTH COF was dispersed in dimethylformamide via ultrasonication. Thermoplastic polyurethane (TPU) was then added at a COF-to-TPU weight ratio of 1:20, and the mixture was stirred vigorously for 12 hours to obtain a homogeneous 1 wt% COF spinning solution. The solution was electrospun using a

commercial electrospinning apparatus under the following parameters: positive voltage of 16 kV, negative voltage of 2.5 kV, injection rate of 1 mL/h, and collector rotation speed of 300 rpm.

Evaluation of Antibacterial Activity of COF/TPU Membranes

The antibacterial performance of the photocatalytic COF/TPU membranes under visible light irradiation was evaluated according to GB/T 30706-2014 and GB 4789.2-2022 with minor modifications. Briefly, 100 μL of bacterial suspension (10^6 CFU/mL) was applied onto a 20 mm × 20 mm electrospun membrane and covered with a medical-grade polyethylene (PE) film (0.02-0.1 mm thick) to ensure uniform distribution. The assembly was then exposed to simulated solar irradiation for 30 minutes. After irradiation, the membrane was thoroughly rinsed with 10 mL of sterile saline. From the resulting eluate, 9 mL was discarded and the remaining 1 mL was subjected to a two-fold serial dilution in sterile saline. A 100 μL aliquot of the diluted solution was spread on nutrient agar plates and incubated at 37 °C for 20 h. Bacterial colonies were enumerated using the standard plate count method, and the antibacterial rate was calculated using Equation (1). A material-free negative control was processed identically. All experiments were performed in triplicate, and data are expressed as mean ± standard error.

Evaluation of Antiviral Activity of COF/TPU Membranes

A viral stock solution was diluted 10-fold, and 200 μL of the suspension was applied to each sample. A xenon lamp (110 mW/cm^2) was used to irradiate each sample for 30 minutes. After irradiation, the samples were rinsed three times with 1 mL PBS. MDCK cells were seeded in 96-well plates at a density of 1.5 × 10^6 cells/mL and cultured for 24 h. The virus suspension recovered from the treated samples was serially diluted 10-fold in basic DMEM from 10^{-1} to 10^{-7}. After cell confluence was reached, the monolayers were washed twice with PBS and inoculated with 100 μL/well of diluted virus suspension (N = 3). Control wells included normal cells (100 μL DMEM/well) and a virus control (untreated virus + DMEM). The plates were incubated for 2 h, washed twice with PBS, replenished with DMEM, and cultured for another 72 h. Cytopathic effects (e.g., cell shrinkage, rounding, and detachment) were recorded. The $TCID_{50}$ was calculated using the Reed–Muench method [Equations (3) and (4)], and the antiviral rate was determined with Equation (5):

$$\text{Infection Rate (\%)}=\frac{C_P}{C_P+C_N}\times 100\% \quad (3)$$

Among them, Cumulative Positives (C_P) represents the sum of infected wells from the most concentrated dilution (lowest exponent) to the current dilution, Cumulative Negatives (C_N) represents the sum of non-infected wells from the most dilute dilution (highest exponent) to the current dilution.

$$\log_{10}(TCID_{50})=\log_{10}(D_a)-\frac{P_a-50\%}{P_a-P_b}\times\log_{10}(S) \quad (4)$$

Among them, D_a represents the dilution factor with infection rate(%) ≥ 50%， P_a represents the cumulative infection rate at Da， P_b represents the cumulative infection rate at the next higher dilution (which is ≤ 50%), S represents the dilution factor step between consecutive dilutions.

$$\text{Antiviral activity rate (\%)}=[1-\frac{10^{\log_{10}\mathrm{TCID}_{50}^{(\mathrm{treated})}}}{10^{\log_{10}\mathrm{TCID}_{50}^{(\mathrm{control})}}}]\times 100\% \qquad (5)$$

Among them, represents the $TCID_{50}$ of the experimental group treated with the material, while $\mathrm{TCID}_{50}^{(\mathrm{control})}$ represents the $TCID_{50}$ of the control group.

Antibacterial Performance Evaluation of Functionalized Masks

The outer layer of a commercial N95 mask was carefully cut and flattened. A prepared electrospun membrane (CuMet-TP COF/TPU or CuMet-BTTH COF/TPU) was inserted as the second functional layer, gently placed between the original first and third layers of the mask, and secured with a clip. An aerosolized *E. coli* suspension (10^7 CFU/mL) was sprayed toward the mask surface for 5 minutes at a flow rate of 0.2 mL/min, with the atomizer outlet positioned 10 cm away. The functionalized mask was subsequently irradiated under simulated sunlight for 30 minutes. Each layer was then thoroughly rinsed with 20 mL of sterile saline. A 100 μL aliquot of the rinse solution was spread on nutrient agar plates and incubated at 37 °C for 20 h. Bacterial concentration was quantified using the standard plate count method, and the antibacterial rate was calculated according to Equation (1). To evaluate residual or adherent bacteria, each rinsed layer was further incubated on LB agar at 37 °C for 20 h. Commercial N95 masks without functional modification were tested under identical conditions as negative controls. All experiments were performed in triplicate, and results are expressed as mean ± standard error.

S3. Structural Modeling

Table S1. Fractional atomic coordinates for the unit cell of CuMet-TP COF with ABC packing.

Name	CuMet-TP	Space Group	R-3
a (Å)	25.8615	b (Å)	25.8615
c (Å)	14.4211	α	90
β	90	γ	120
Unit Cell Volume (Å^3)		8352.88	
Atom Name	x	y	z
Cu1	-0.33333	-0.16667	0.33333
O1	0.40023	-0.38351	0.28731
N1	0.30539	-0.49069	0.29004
N2	0.57823	-0.11202	0.29465
N3	0.58146	-0.20131	0.31399
N4	-0.32133	-0.08666	0.31926
N5	-0.32124	-0.32169	0.3715
C1	0.36898	-0.36038	0.28735
C2	0.306	-0.39639	0.28767
C3	0.27668	-0.45905	0.29115
C4	0.55081	-0.17239	0.29906
C5	0.6371	-0.07062	0.30306
C6	-0.27824	-0.34114	0.3899
C7	0.61635	-0.36774	0.36224

Table S2. Fractional atomic coordinates for the unit cell of CuMet-BTTH COF with ABC packing.

Name	CuMet-BTTH	Space Group	R-3
a (Å)	50.5801	b (Å)	50.5801
c (Å)	16.8219	α	90
β	90	γ	120
Unit Cell Volume (Å^3)		37270.5	
Atom Name	x	y	z
Cu1	0.66667	0.83333	0.33333
O1	1.30283	0.4885	0.15765
N1	1.22614	0.42766	0.28472
N2	1.61657	0.84358	0.23444
N3	1.62242	0.81109	0.32543
N4	0.66929	0.86404	0.25916
N5	0.6806	0.77776	0.4947
C1	1.60469	0.81831	0.28131
C2	1.64619	0.86531	0.22234
C3	1.64926	0.75392	0.50965
C4	0.7047	0.77541	0.53571
C5	1.34535	0.69828	0.26946
C6	1.36494	0.68627	0.26962
C7	1.29364	0.69099	0.26637
C8	1.42456	0.72325	0.26286
C9	1.25662	0.71356	0.25828
C10	1.46881	0.76932	0.20998
C11	1.50036	0.78904	0.20528
C12	1.52025	0.7828	0.24927
C13	1.50814	0.75668	0.29757
C14	1.47661	0.73705	0.30204
C15	1.19646	0.74989	0.24462

S4. Molecular dynamics simulation

Atomistic molecular dynamics simulations have been performed in the GROMACS[1] (version 2022.6) simulation package, using the Amber14sb force field for the lipids and the General Amber force field (GAFF2) for the COF material as well as the TIP3P water model. The interaction potentials V between atoms include the bonded and non-bonded terms, as described in Equation (2).

$$V=V_{bonded}+V_{nonbonded} \tag{2}$$

The bonded terms include the harmonic oscillation of bonds and angles as well as the torsional rotation of dihedrals (Equation 3); the non-bonded term include the electrostatic interactions between atoms with partial charges as well as the Van der Waals interactions described through the Lennard-Jones 12-6 potentials [Equation (4)].

$$V_{bonded}=\sum_{bonds}\frac{1}{2}k_b(l\text{-}l_0)^2+\sum_{angles}\frac{1}{2}k_\theta(\theta\text{-}\theta_0)^2+\sum_{torsions}k_\phi[1+cos(n\phi\text{-}\phi_0)] \tag{3}$$

$$V_{nonbonded}=\sum_{i=1}^{N}\sum_{j=i+1}^{N}\left\{\frac{q_iq_i}{4\pi\epsilon_0\epsilon_r r_{ij}}+4\varepsilon_{ij}\left[\left(\frac{\sigma_{ij}}{r_{ij}}\right)^{12}\text{-}\left(\frac{\sigma_{ij}}{r_{ij}}\right)^{6}\right]\right\} \tag{4}$$

The lipid bilayer composed of 576 POPE, 108POPG and 36 PVCL2 were composed at the initial size of 15 nm and equilibrated for 20 ns at the final size of around 14.7×14.7 nm. The unit-cell of the COFs structure are replicated to the sizes around 11×11×3 nm. The materials were then separately placed on top of the lipid bilayer with initial system size around 14.7×14.7×17 nm, and thousands of steps of energy minimization were performed before addition of more than 50000 water molecules. After thousands of steps of energy minimization, the systems were equilibrated for 2 ns with restraints on the lipid headgroups and the heavy atoms in the materials to relax the water concentration. The production runs extended for another 20 ns under the NPT ensemble. The temperature was coupled using the Nose-Hoover method and the pressure was coupled to 1 atm using the Parrinello-Rahman method. The cutoff scheme of 1.2 nm was implemented for the non-bonded interactions, and the Particle Mesh Ewald method[2] with a Fourier spacing of 0.1 nm was applied for the long range electrostatic interactions. All covalent bonds with hydrogen atoms were constraint using the LINCS algorithm[3].

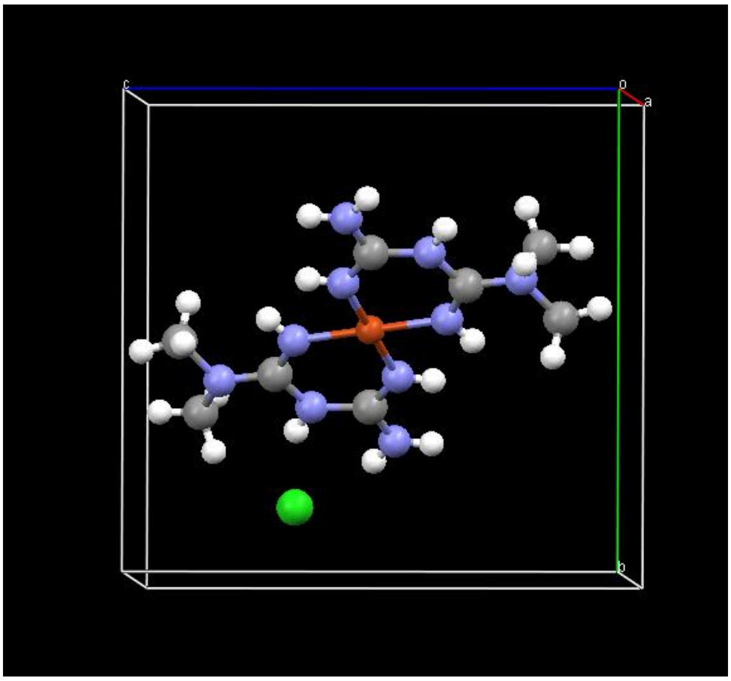

Figure S1. The unit cell structure of CuMet.

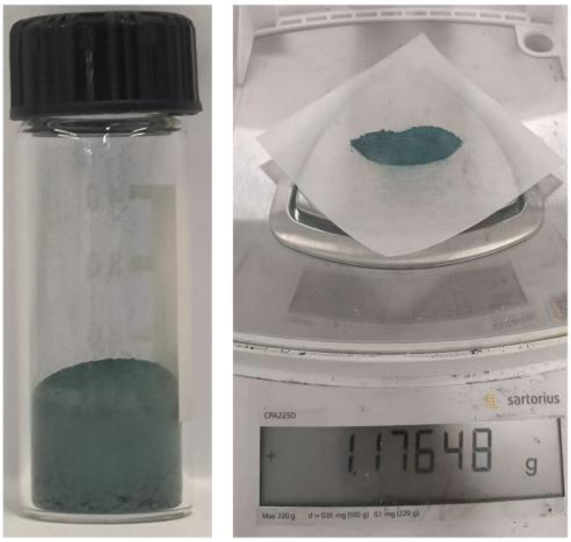

Figure S2. Photograph of as-synthesized CuMet-TP COF (gram-scale).

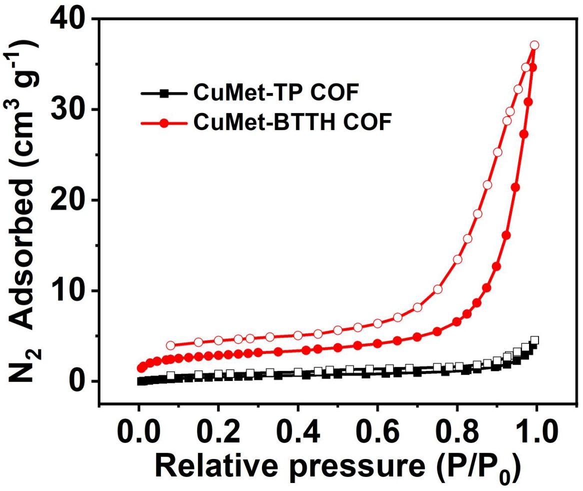

Figure S3. Nitrogen adsorption-desorption curves for CuMet-TP COF and CuMet-BTTH COF.

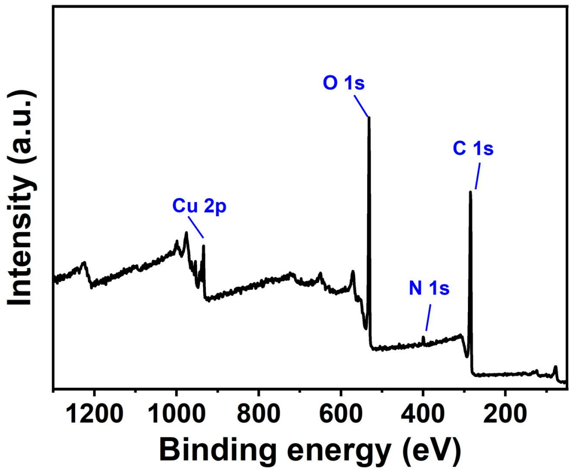

Figure S4. XPS spectra of CuMet-TP COF.

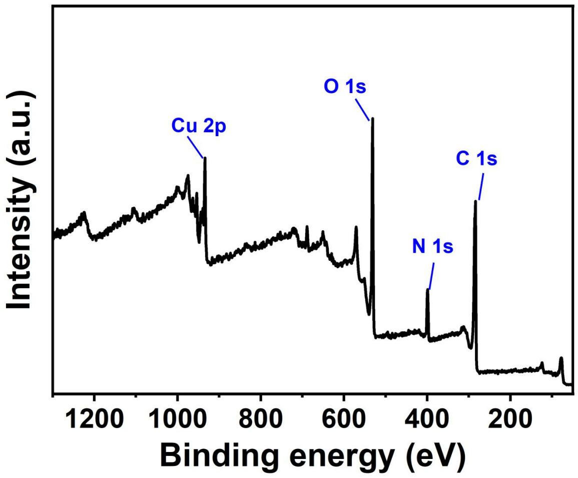

Figure S5. XPS spectra of CuMet-BTTH COF.

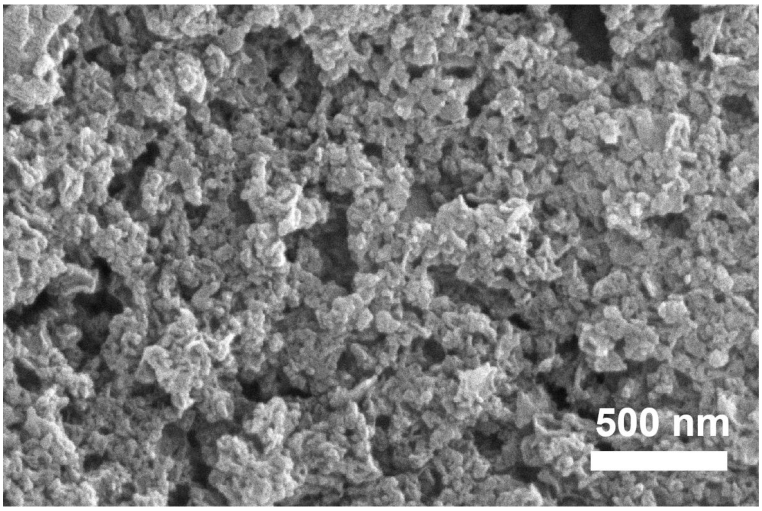

Figure S6. SEM image of CuMet-TP COF.

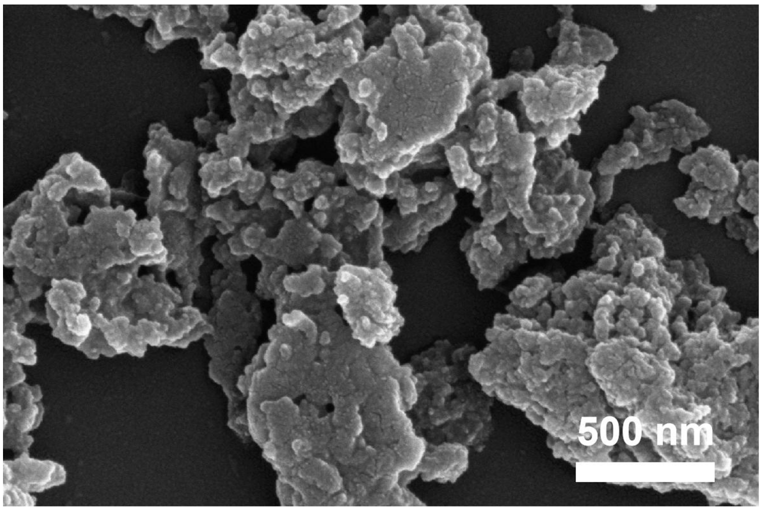

Figure S7. SEM image of CuMet-BTTH COF.

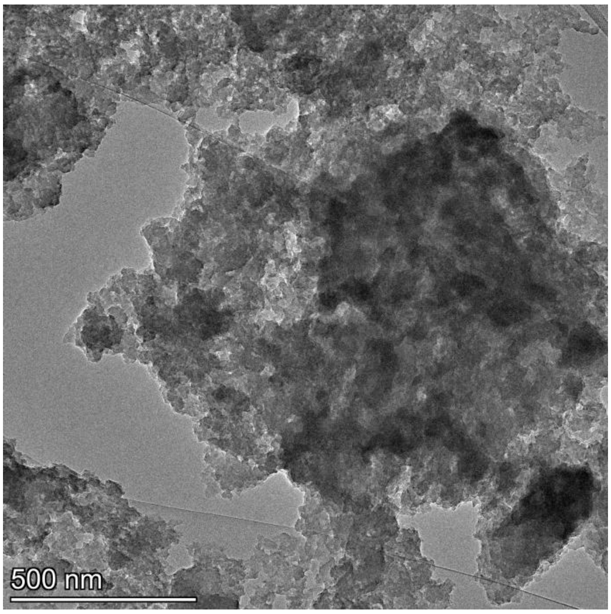

Figure S8. TEM image of CuMet-TP COF.

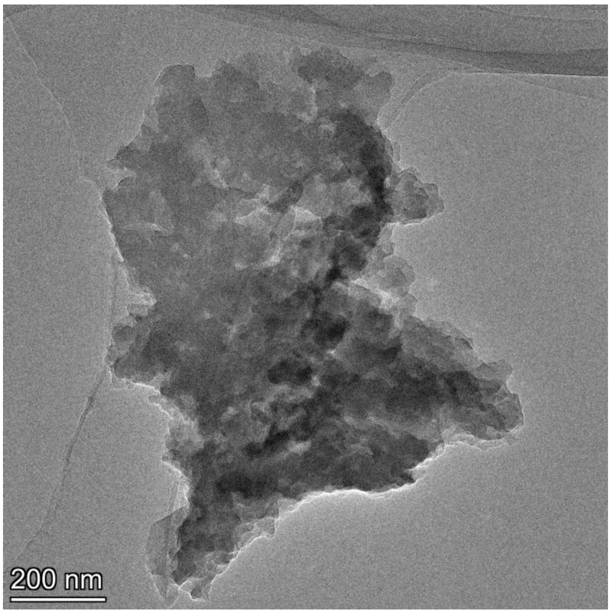

Figure S9. TEM image of CuMet-BTTH COF.

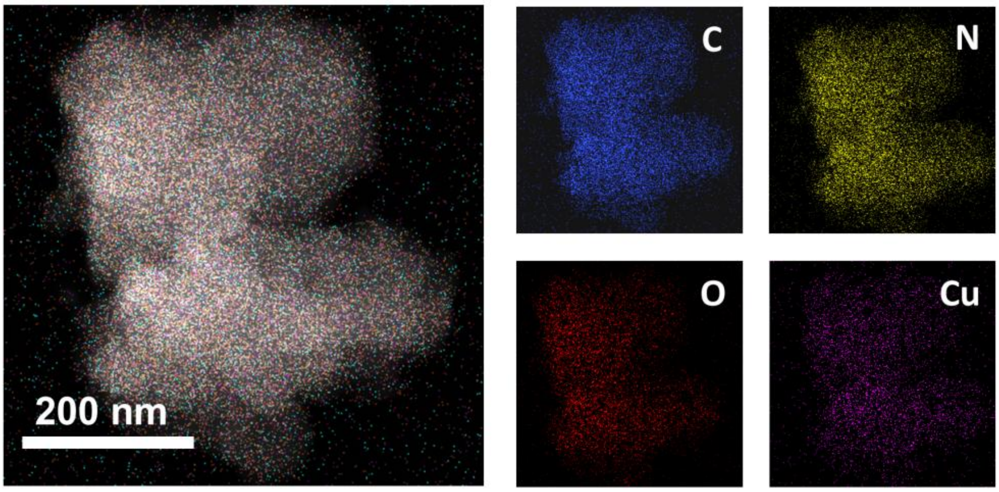

Figure S10. Element mapping images of CuMet-BTTH COF.

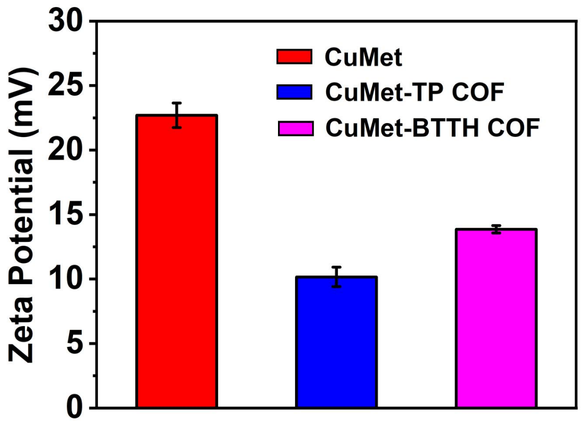

Figure S11. Zeta potentials of CuMet, CuMet-TP COF , and CuMet-BTTH COF.

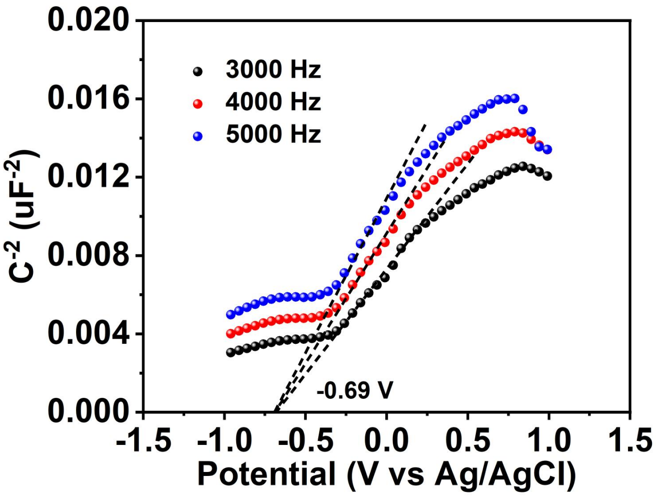

Figure S12. Mott–Schottky plot of CuMet-TP COF.

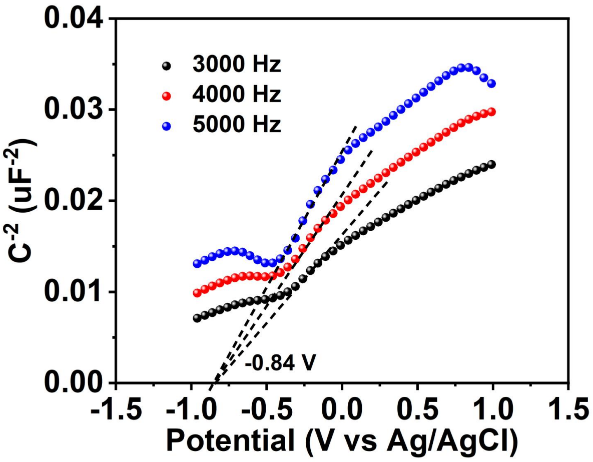

Figure S13. Mott–Schottky plot of CuMet-BTTH COF.

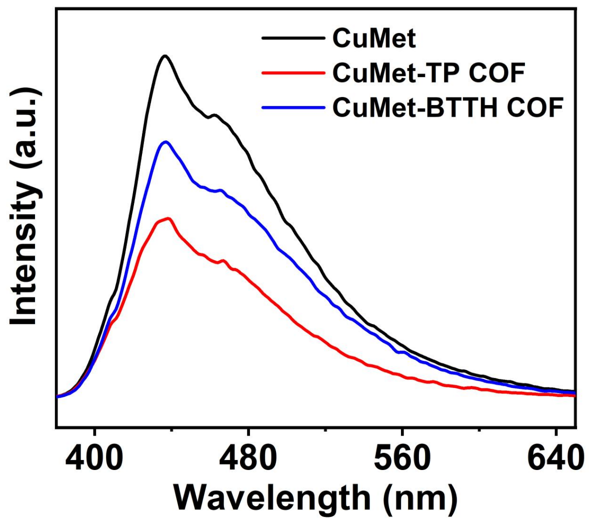

Figure S14. Photoluminescence (PL) spectra of CuMet, CuMet-TP COF, and CuMet-BTTH COF.

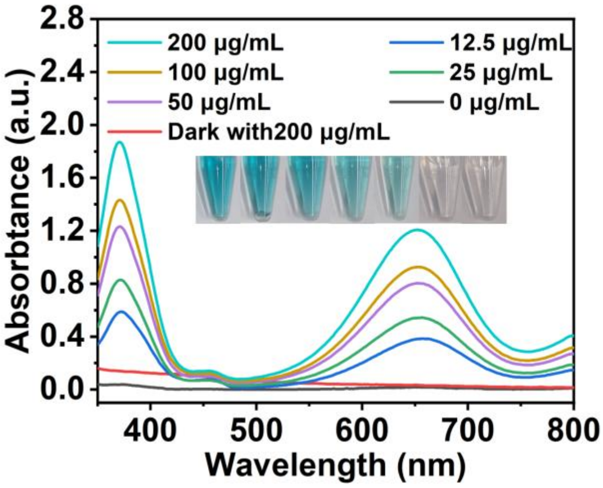

Figure S15. Photocatalytic oxidation of TMB by CuMet-TP COF at different concentrations.

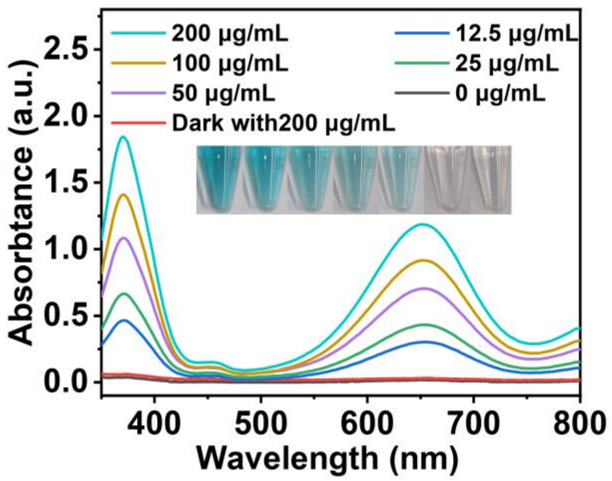

Figure S16. Photocatalytic oxidation of TMB by CuMet-BTTH COF at different concentrations.

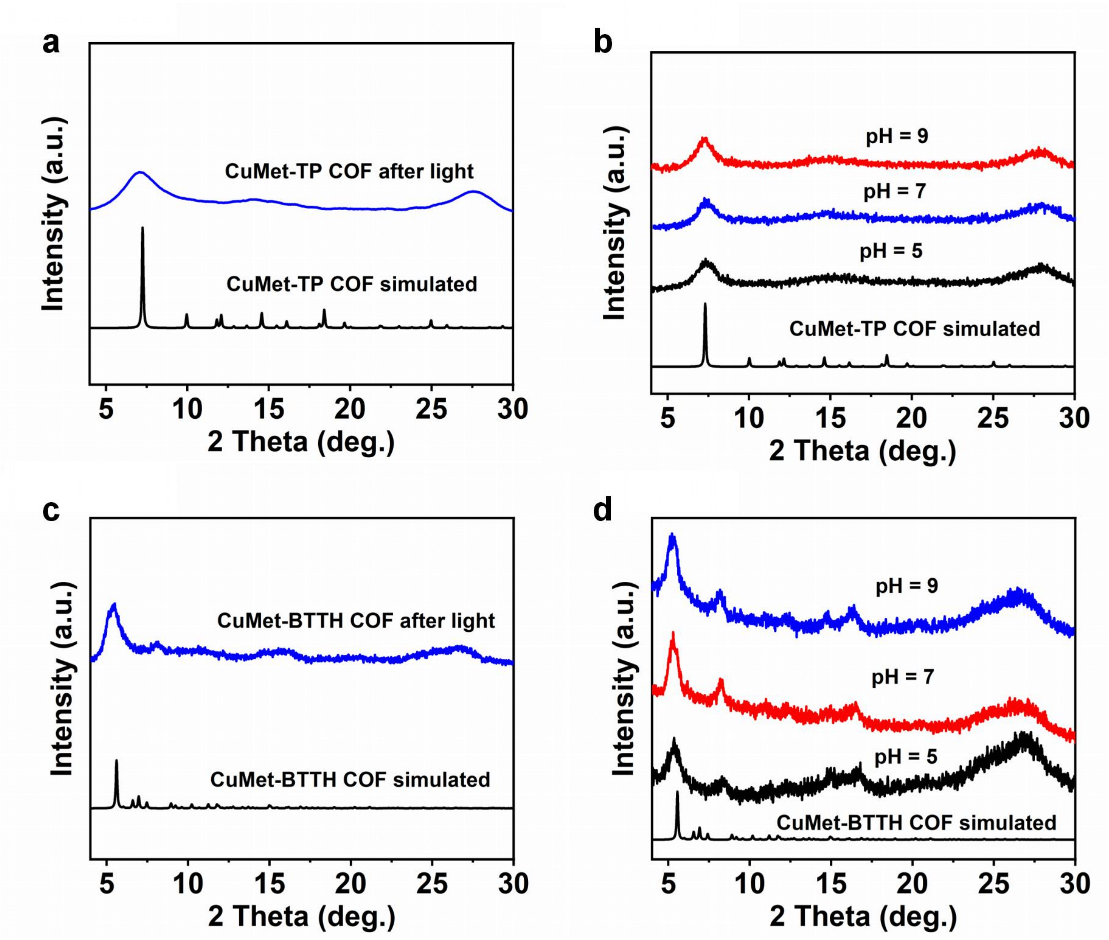

Figure S17. Stability of CuMet-TP and CuMet-BTTH COFs. XRD patterns of (a) CuMet-TP COF and (c) CuMet-BTTH COF after light irradiation. XRD patterns of (b) CuMet-TP COF and (d) CuMet-BTTH COF after soaking at the solution with different pH for 24 h.

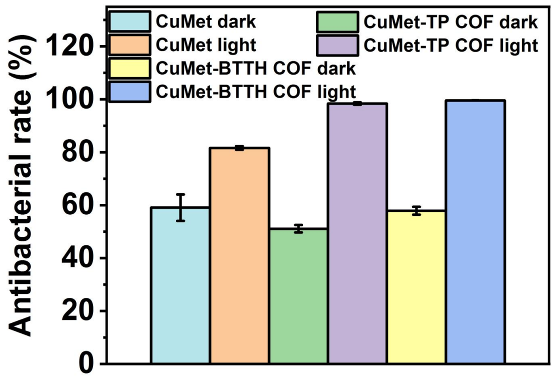

Figure S18. Antibacterial rates against *S.aureus* (50 μg/mL , 30 min dark/light exposure): CuMet, CuMet-TP COF, and CuMet-BTTH COF.

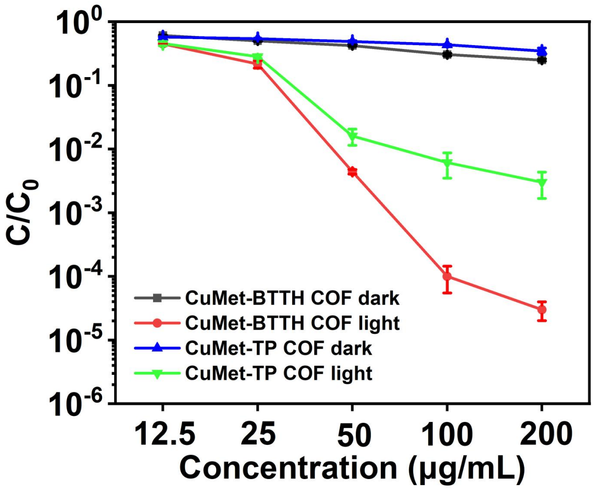

Figure S19. Antibacterial activity of CuMet-TP COF and CuMet-BTTH COF against *S.aureus* under light/dark conditions at different concentrations.

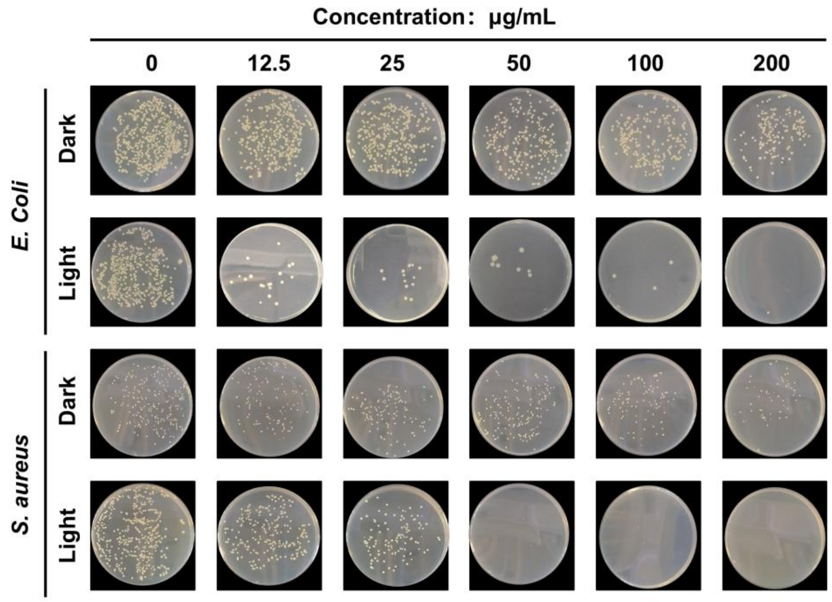

Figure S20. Photographs of *E.coli* and *S.aureus* formed on LB agar plates after different treatments of CuMet-TP COF.

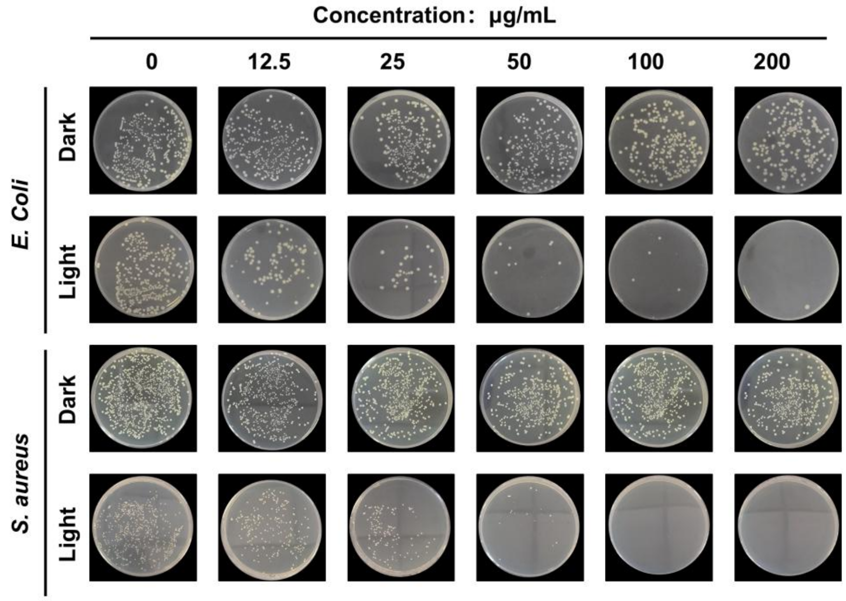

Figure S21. Photographs of *E.coli* and *S.aureus* formed on LB agar plates after different treatments of CuMet-BTTH COF.

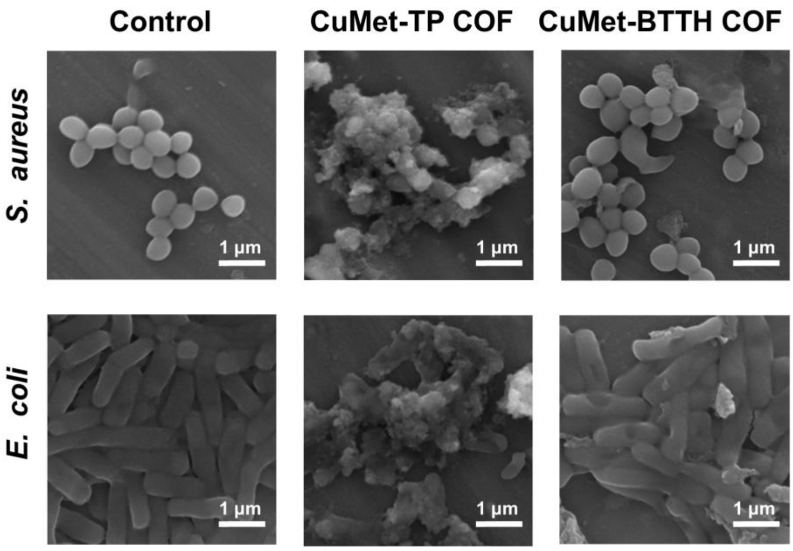

Figure S22. SEM images of *E.coli* and *S.aureus* under various treatments (scale bar, 1 μm).

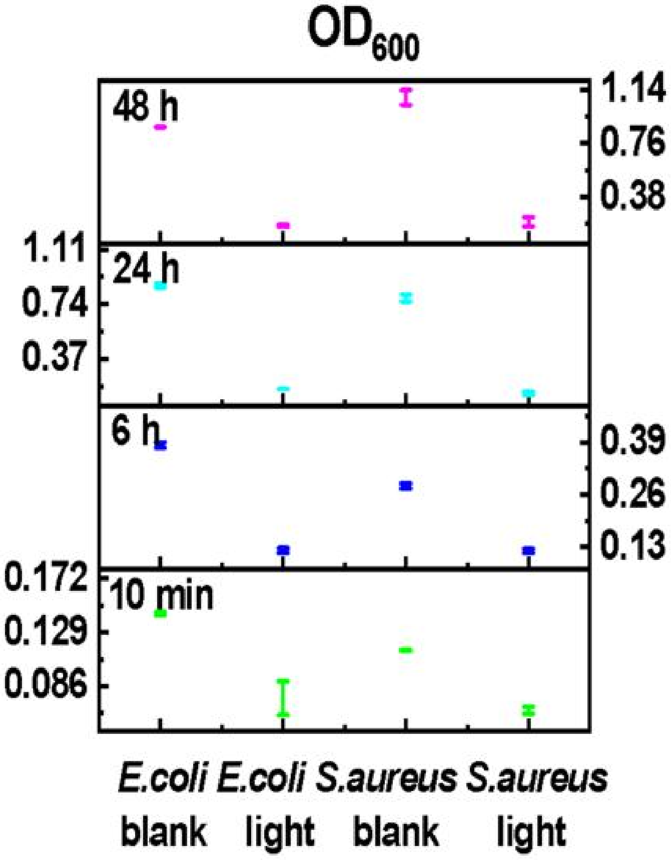

Figure S23. Bacterial regrowth was measured within 2 days after different treatments with CuMet-TP COF.

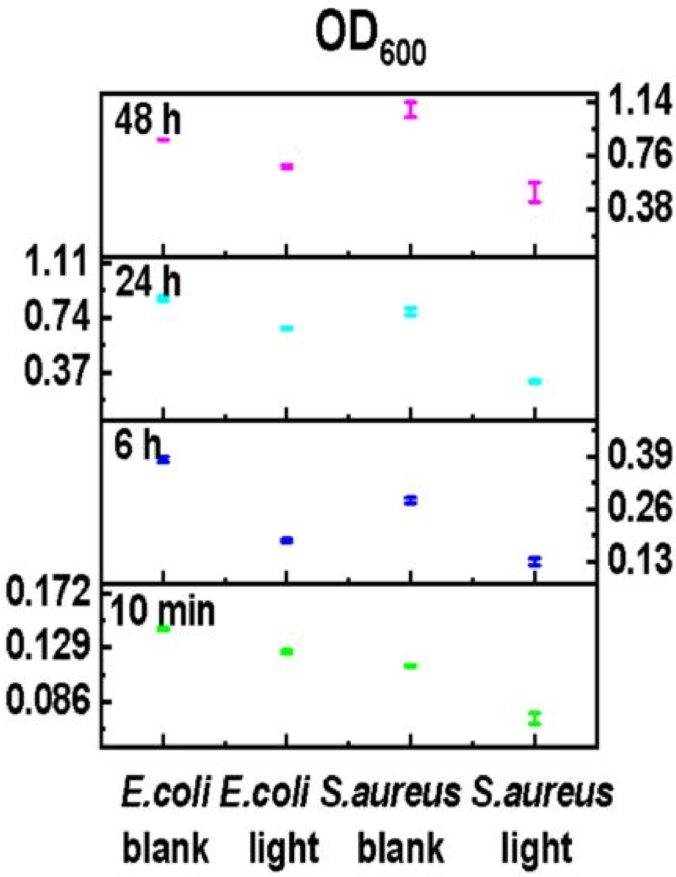

Figure S24. Bacterial regrowth was measured within 2 days after different treatments with CuMet-BTTH COF.

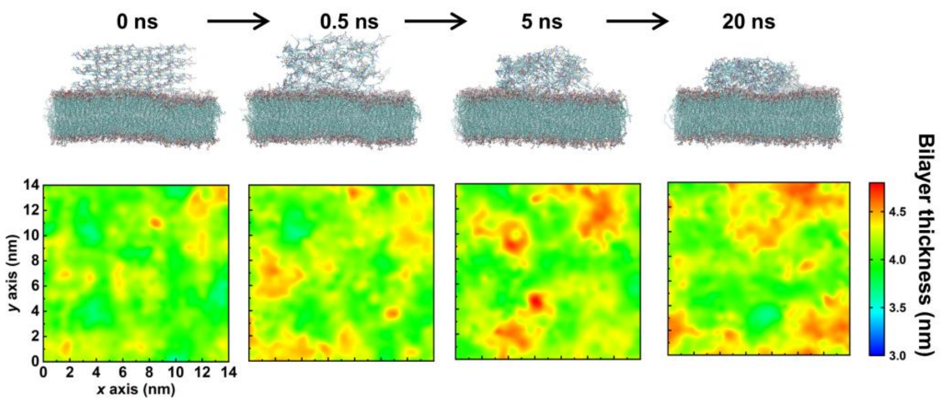

Figure S25. Molecular dynamics simulation process of interaction between CuMet-BTTH COF and *E.coli* bacterial phospholipid membrane within 20 ns.

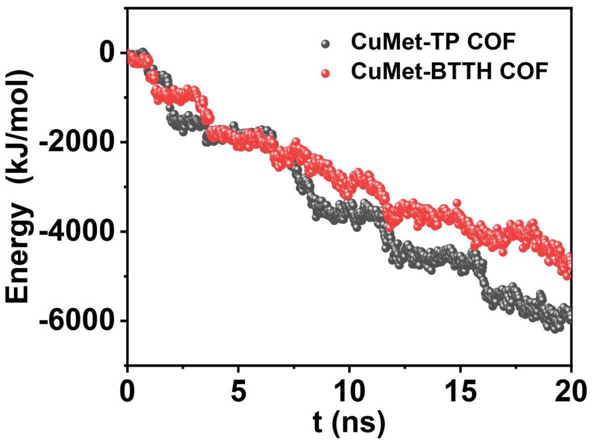

Figure S26. The coulomb force statistics of metformin-based COFs and phospholipid membrane during kinetic simulation.

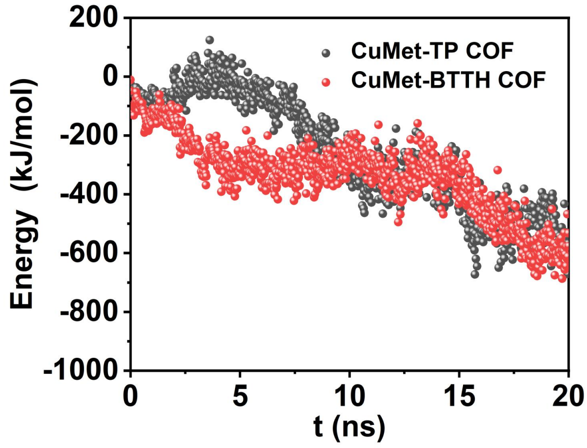

Figure S27. The Van der Waals force statistics of metformin-based COFs and phospholipid membrane during kinetic simulation.

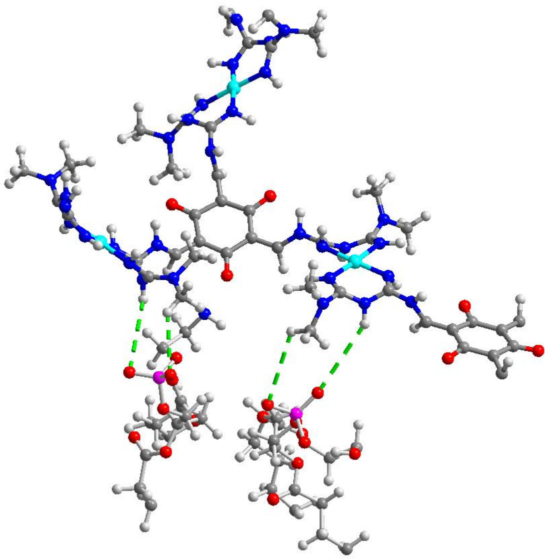

Figure S28. The intermolecular hydrogen bond interaction between CuMet-TP COF and the phospholipid membrane at 0.5ns.

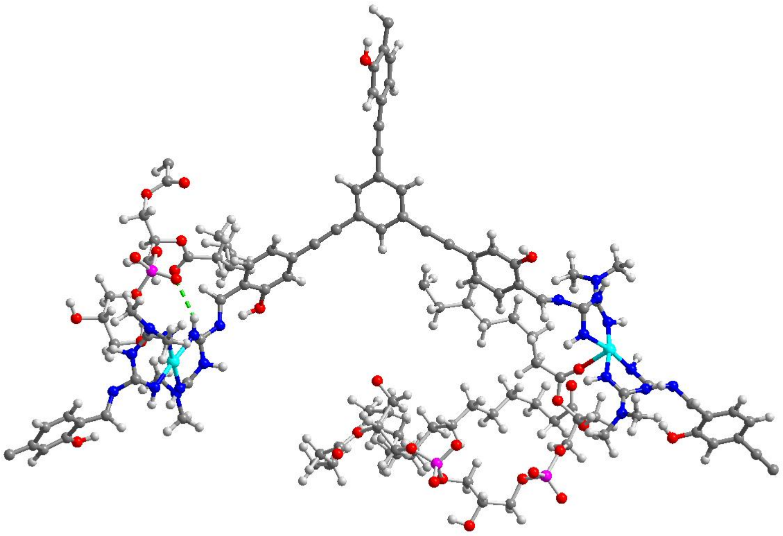

Figure S29. Intermolecular interactions of hydrogen bonds and coordination between CuMet-BTTH COF and phospholipid membranes at 0.5ns.

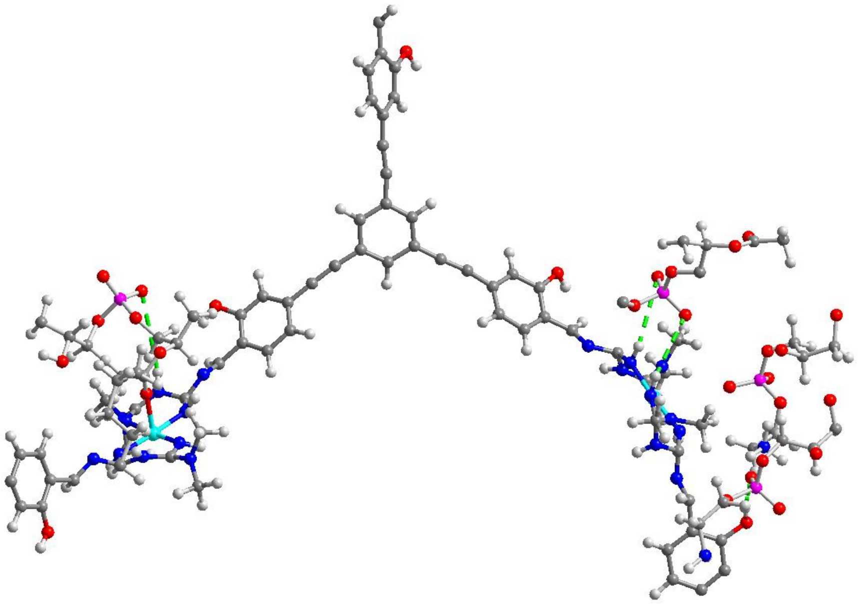

Figure S30. Intermolecular interactions such as hydrogen bonds and coordination between CuMet-BTTH COF and phospholipid membranes at 20ns.

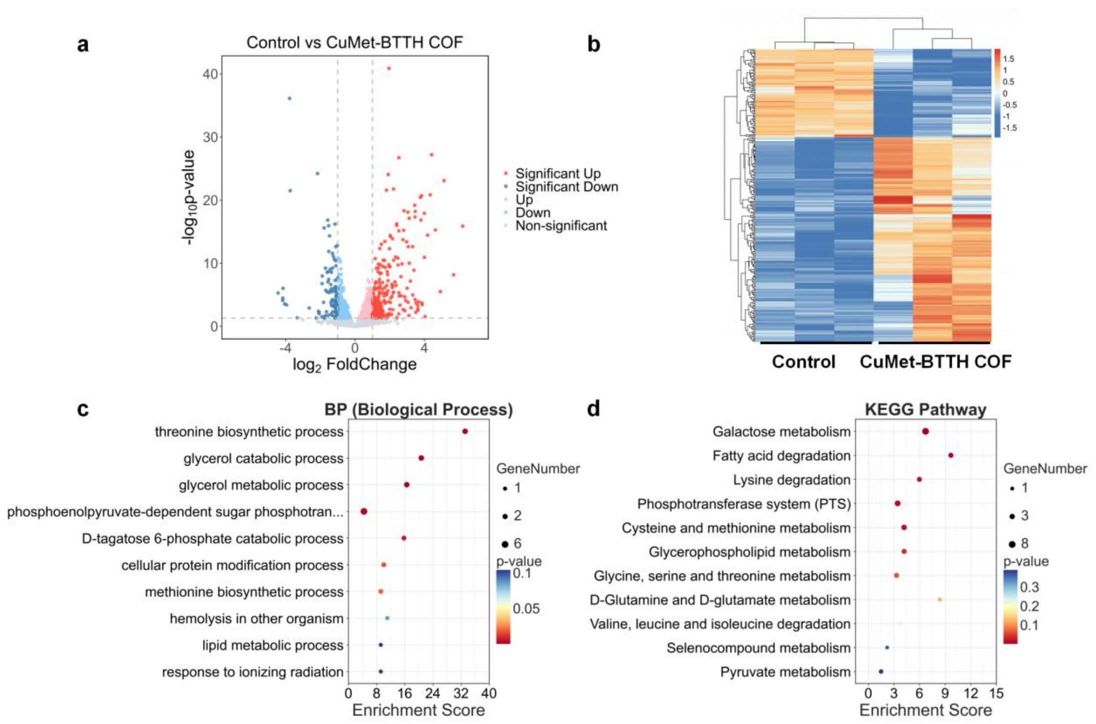

Figure S31. (a) Volcano plot analysis of different expression of genes in CuMet-BTTH COF group and control group; (b) Heatmap analysis of genes differentially expressed in CuMet-BTTH COF group and control group; (c-d) GO and KEGG analysis of genes differentially expressed in CuMet-BTTH COF group and control group.

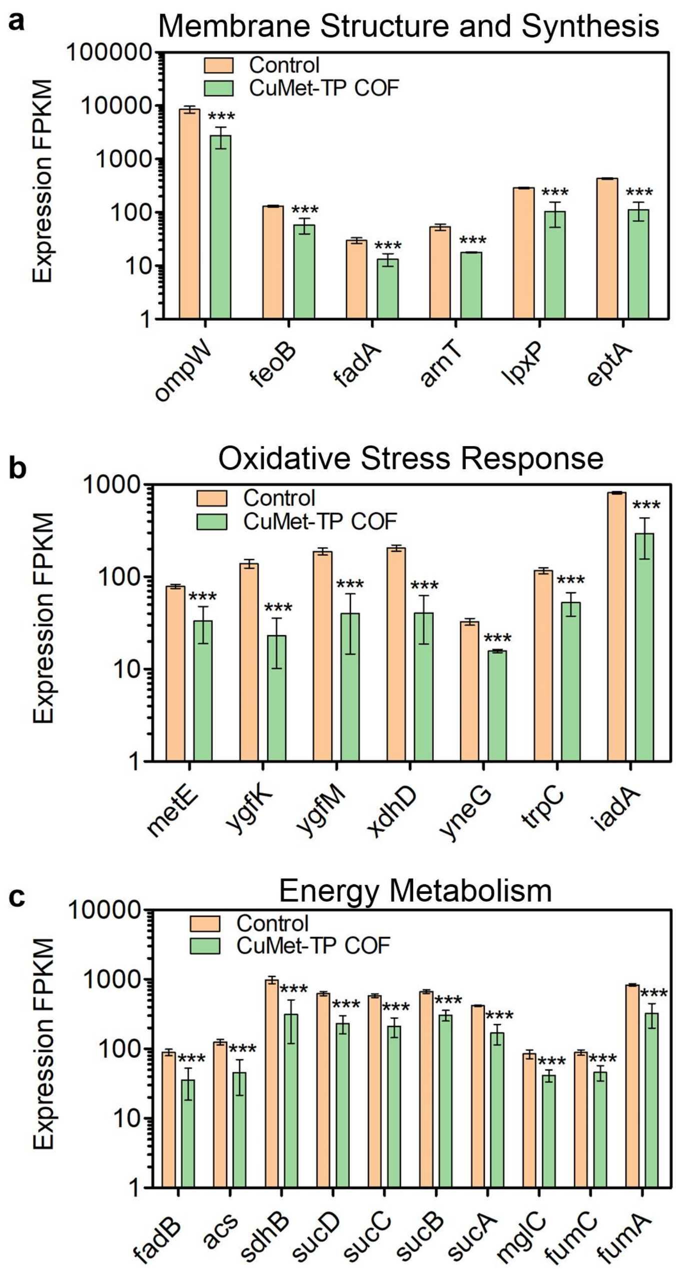

Figure S32. The expression of genes related to (a) membrane structure and synthesis, (b) oxidative stress response, (c)energy metabolism in CuMet-TP COF group and control group.

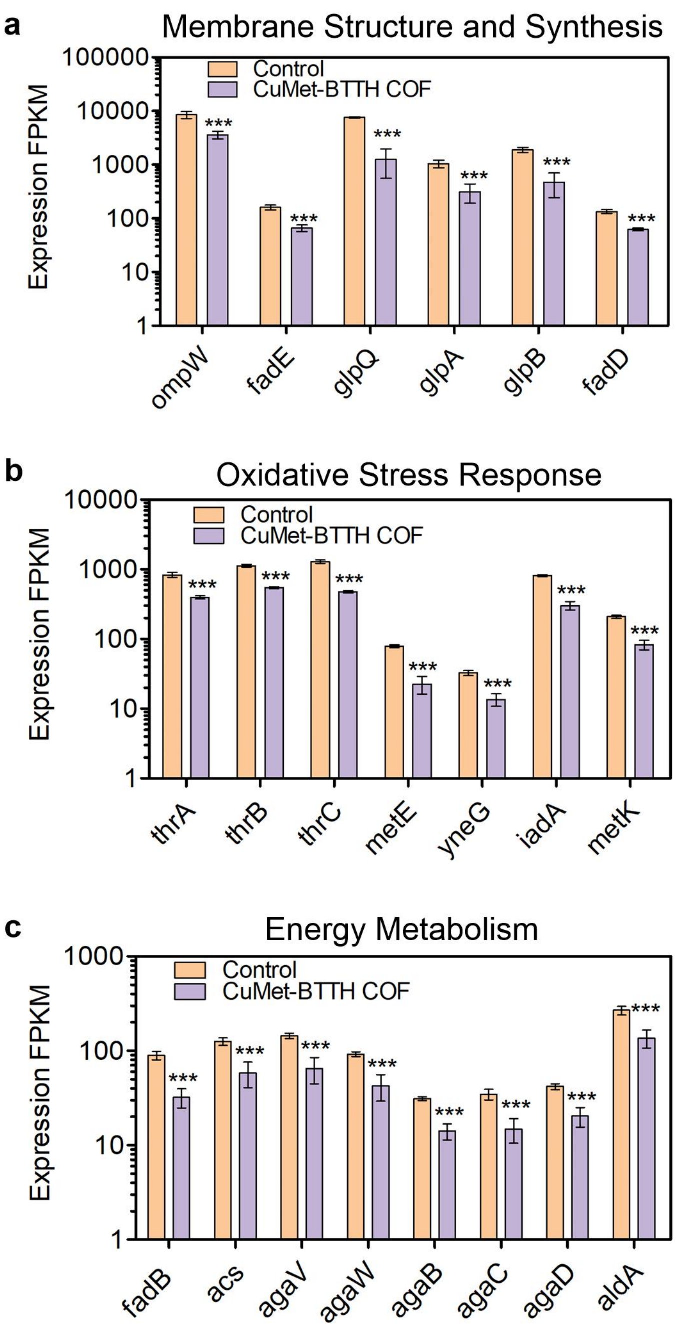

Figure S33. The expression of genes related to (a) membrane structure and synthesis, (b) oxidative stress response,(c) energy metabolism in CuMet-BTTH COF group and control group.

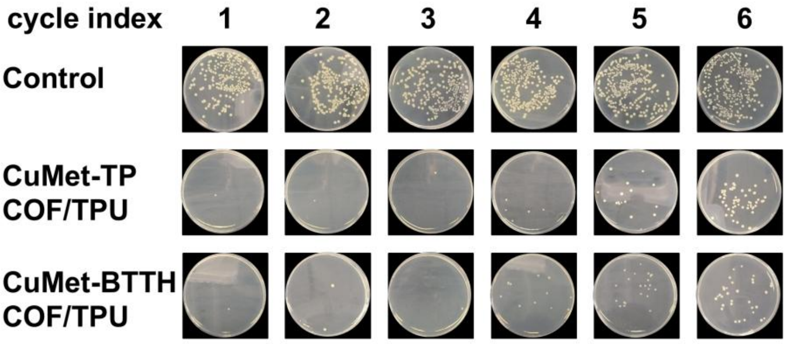

Figure S34. Photographs of *E.coli* colonies formed on LB agar plates when different COF/TPU composite membranes were recycled for 6 times.

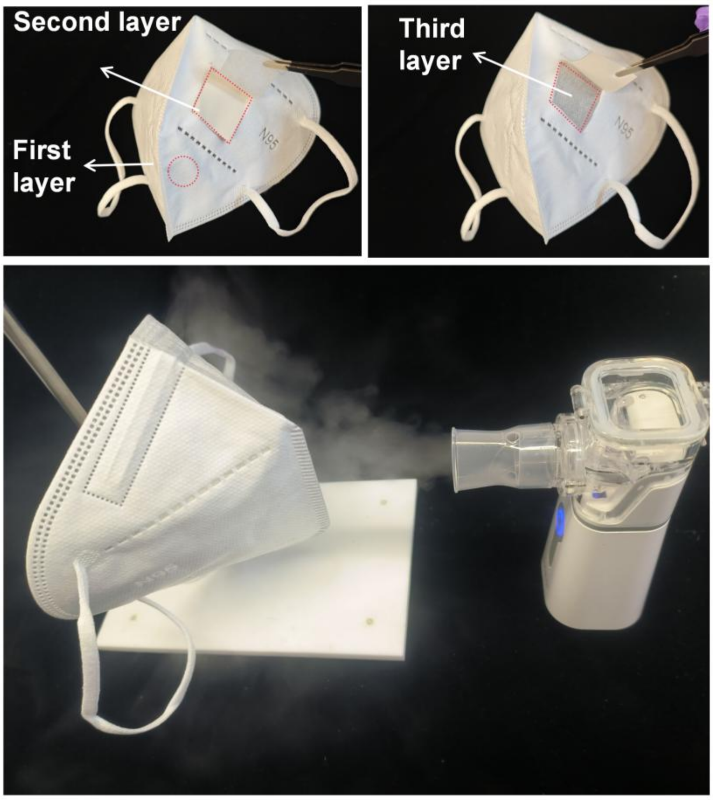

Figure S35. The optical image and bioaerosol generation device of each layer in the three-layer N95 mask based on metformin-based MCOFs/TPU.

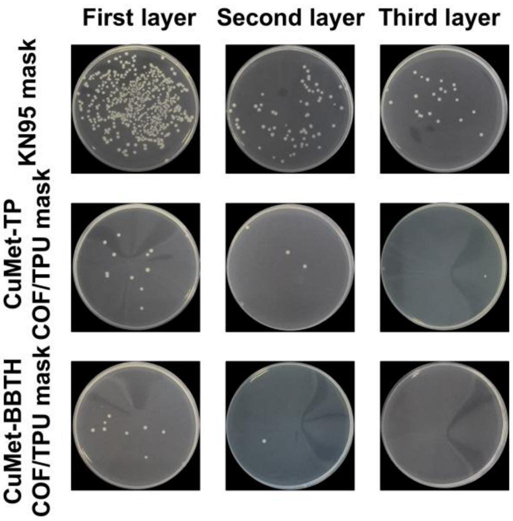

Figure S36. Photographs of *E.coli* colonies formed by diluted suspensions of the 1st, 2nd, and 3rd layers from photocatalytically treated COF/TPU composite mask membranes on LB agar plates.

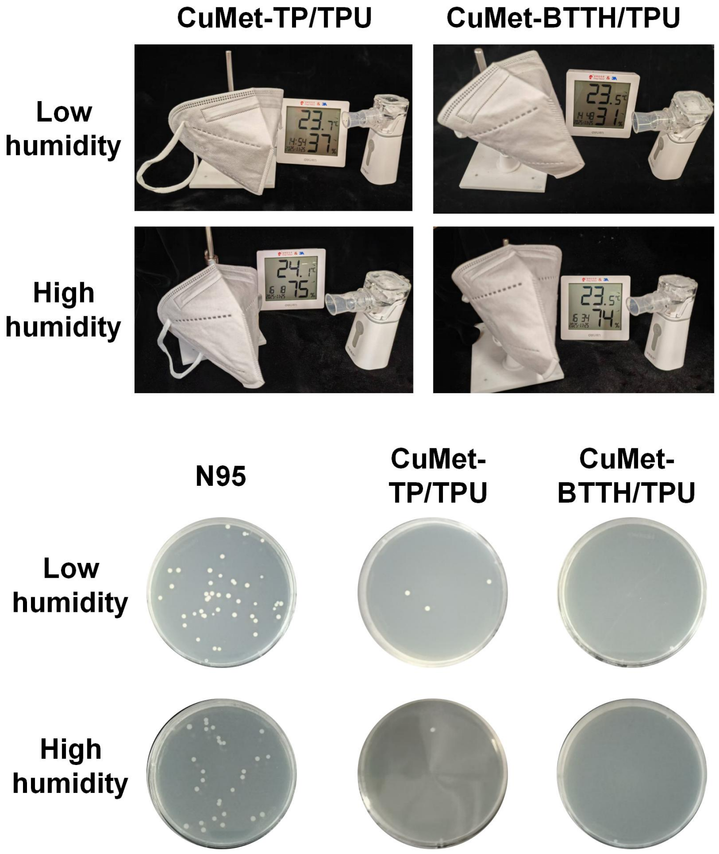

Figure S37. The antibacterial activity of masks assembled with CuMet-TP COF/TPU or CuMet-BTTH COF/TPU films under different humidity conditions. Photographs of *E.coli* colonies formed by diluted suspensions of the 2nd layer from photocatalytically treated COF/TPU composite mask membranes on LB agar plates.

Table S3 The copper concentration in the supernatant after the antibacterial experiments.

Sample	Cu element content（μg/L)	Mean value（ng/mL)
	9.9183	
CuMet-TP COF	10.2183	10.1
	10.1880	
	101.2621	
CuMet-BTTH COF	102.3914	102
	101.5151	

Table S4 Hematological parameter tables related to the CuMet-TP COF group and the CuMet-BTTH COF group compared with the control group.

Group	Parameter	Value	Reference Range
**Control**	ALT (U/L)	45.34±3.485	10.06-96.47
	AST (U/L)	117.6±8.095	36.31-235.48
	UREA (mg/dL)	24.38±0.8125	10.81-34.74
	CREA (μmoL/L)	44.70±2.099	10.91-85.09
**CuMet-TP COF**	ALT (U/L）	39.24±2.822	10.06-96.47
	AST (U/L)	122.0±9.026	36.31-235.48
	UREA (mg/dL)	27.09±2.545	10.81-34.74
	CREA (μmoL/L)	36.75±2.846	10.91-85.09
**CuMet-BTTH COF**	ALT (U/L）	38.69±4.533	10.06-96.47
	AST (U/L)	113.9±3.632	36.31-235.48
	UREA (mg/dL)	17.82±0.8087	10.81-34.74
	CREA (μmoL/L)	36.36±1.361	10.91-85.09

Table S5 A comparison table of the performance, safety and potential cost of metformuan-based COFs with other reported antibacterial COFs/ MOFs.

**Materials**	**Antibacterial activity**	**Safety**	**Potential cost RMB/g**	**Ref.**
**CuMet-TP COF**	12.5 ug/mL<br>>96% *E. coli* (30min light)<br>200 ug/mL<br>~100% *E. coli* (30min light)	LD_{50}> 5g/kg	**~50**	**This work**
ZIF-8	300 ug/mL<br>~96% *E. coli* (30min light)<br>500 ug/mL<br>~100% *E. coli* (2h light)	–	–	4
Cu/Zn－MOF @GOx	40 ug/mL<br>>95.05% *E. coli*	At 40 µg/mL, L929 cells grew without notable material cytotoxicity	–	5
Ag-MOF	MIC: 8 µg/mL	From 0 to 160 µg/mL, showed no cytotoxic effects on HUVECs and MLE12 cells	–	6
ZAIS QDs@ZIF-8	300 ug/mL<br>~100% *E. coli* (1h light)	–	–	7
2D ZnTCPP	300 ug/mL<br>~100% *E. coli* (1h light)	-	–	8
GZHMU-2	60 ug/mL<br>~99% *E. coli* (30min light)	–	–	9

References

1. Hess, B., et al., *J. Chem. Theory Comp.*, 2008, **4**, 435.
2. Essmann, U., et al., *J. Chem. Phys.*, 1995, **103**, 8577.
3. Hess, B., et al., *J. Comput. Chem.*, 1997, **18**, 1463.
4. Li, P., et al., *Nat. Commun.* 2019, **10** , 1.
5. Peng, L., et al., *Nanoscale* 2022, **14**, 2052.
6. Huang, R., et al., *J. Nanobiotechnology* 2021, **19**, 299.
7. Wang, M., et al., *Chem. Eng. J.* 2021, **426**, 130832.
8. Zhang, L., et al., *Angew. Chem. Int. Ed.* 2021, **6**, 22664.
9. Wang, J., et al., *Adv. Mater.* 2024, **36**,2311519.
